# Synthesis and structure of (NH_4_)_12_[Mo_36_O_112_(OH)_4_(H_2_O)_10_], the first example of the [Mo_36_O_112_]^8−^ molybdate anion cluster to bear a charge of 12−

**DOI:** 10.1107/S2056989026010388

**Published:** 2026-10-13

**Authors:** Rajpal S. Gaur, Stephanie L. Brock, Cassandra L. Ward

**Affiliations:** a GGP Nano Materials, LLC, 2611 Parmenter Blvd., Royal Oak, Michigan 48073, USA; b Department of Chemistry Wayne State University, 5101 Cass Avenue Detroit Michigan 48202 USA; c Lumigen Instrument Center Wayne State University, 5101 Cass Avenue Detroit Michigan 48202 USA; Universidad de la República, Uruguay

**Keywords:** crystal structure, polyoxometallates, isopolymolybdates, 12− charge, Mo_36_ cluster, ammonium com­pound

## Abstract

Treatment of [Mo_7_O_24_]^6−^ with 0.05 *N* HNO_3_ at pH ∼ 1.8 in the presence of seed crystals of the ammonium salt of Mo-13 isopolymolybdate led to the formation of the title salt, which appears to represent the first reported 12− charged form of this com­position. Analysis of the Mo—O—Mo bond distances obtained from single-crystal X-ray diffraction supports the formulation of the anion in the ammonium com­pound as [Mo_36_O_112_(OH)_4_(H_2_O)_10_]^12−^. Charge balance requires 12 ammonium counter-ions, and the presence of 26 cocrystallized water mol­ecules gives the overall formula (NH_4_)_12_[Mo_36_O_112_(OH)_4_(H_2_O)_10_]·26H_2_O. This structure differs from previously reported Mo_36_ isopolymolybdate phases, including K_8_[Mo_36_O_112_(H_2_O)_16_]·36H_2_O, (H_2_bipy)_4_[Mo_36_O_112_(H_2_O)_16_]·28H_2_O, {(CH_3_NH_3_)_8_[Mo_36_O_112_(H_2_O)_14_]·19H_2_O·2.1(CH_3_NH_3_·NO_3_)}_*n*_, (CH_3_NH_3_)_10_[Mo_36_O_112_(OH)_2_(H_2_O)_14_]·9H_2_O, and K_10_[Mo_36_O_110_(OH)_6_(H_2_O)_12_]·38H_2_O.

## Abstract 2

The single-crystal X-ray structure of (NH_4_)_12_[Mo_36_O_112_(OH)_4_(H_2_O)_10_]·26H_2_O reveals a new ammonium Mo_36_ isopolymolybdate polymorph containing a 12− charged cluster whose formulation is supported by Mo—OH—Mo bond-distance analysis.

## 1. Chemical context

The [Mo^VI^_36_O_112_]^8−^ anionic cluster is a well-established member of the isopolymolybdate family (Gumerova & Rompel, 2020 ▸). The formation of polyoxomolybdates is influenced by acidity, metal concentrations, tem­per­a­ture, counter-cations, and the presence of heteroatoms or other additives. In aqueous solution, [MoO_4_]^2−^, the monomolybdate species present under basic to neutral conditions in alkali-metal molybdate salts such as Na_2_MoO_4_, undergoes dehydrative condensation upon acidification (Sukmana *et al.*, 2024 ▸). The initial solution pH governs the extent of condensation and, consequently, the polyoxomolybdate species formed: lower pH values favor more extensive condensation and the formation of progressively larger clusters, including [Mo_7_O_24_]^6−^, [β-Mo_8_O_26_]^4−^, and ultimately [Mo_36_O_112_(H_2_O)_16_]^8−^ (Gumerova & Rompel, 2020 ▸; Sukmana *et al.*, 2024 ▸; Himeno *et al.*, 1997 ▸; Falaise *et al.*, 2018 ▸).

Paulat-Böschen reported the single-crystal structure of K_8_[Mo_36_O_112_(H_2_O)_16_]·36H_2_O in 1979, describing a potassium salt of the [Mo_36_O_112_]^8−^ cluster that differs structurally from its sodium analogue (Paulat-Böschen, 1979 ▸). Single crystals were obtained at room tem­per­a­ture by mixing equal volumes of 0.2 M K_2_MoO_4_ and 0.4 *N* HNO_3_ solutions. Baizig *et al.* (2018 ▸) later described another potassium-containing Mo_36_ cluster, formulated as K_10_[Mo_36_O_110_(OH)_6_(H_2_O)_12_]·38H_2_O. In that work, single crystals were grown from a heated solution containing K_2_MoO_4_ (3.0 mmol l^−1^) and KNO_3_ (0.5 mmol l^−1^), adjusted to pH 1.8 with 2 M HCl, followed by filtration and slow cooling to room tem­per­a­ture. Atencio *et al.* (2005 ▸) reported the organic–inorganic hybrid phase (H_2_bipy)_4_[Mo_36_O_112_(OH_2_)_16_]·28H_2_O, prepared from an acidified sodium molybdate solution (pH ∼ 1) using 4,4′-bi­pyridine as the organic com­ponent. Related di­ammonium–alkane salts, [NH_2_(CH_2_)_*n*_NH_2_]_4_[Mo_36_O_112_(H_2_O)_*x*_]·*m*H_2_O, have also been reported, where *x* = 14 for *n* = 3–5, *x* = 16 for *n* = 7–11, and *m* ranges from 12 to 32 (Eda & Iriki, 2005 ▸). These phases were crystallized by adding NH_2_(CH_2_)_*n*_NH_2_ and HCl to solutions of the [Mo_36_O_112_]^8−^ ion prepared from aqueous sodium/molybdate using an ion-exchange resin. More recently, Sukmana *et al.* (2024 ▸) dissolved (CH_3_NH_3_)_2_[MoO_4_] in water and adjusted the solution to pH 2 with concentrated nitric acid to obtain single crystals of (CH_3_NH_3_)_10_[Mo_36_O_112_(OH)_2_(H_2_O)_14_]. Although [Mo_36_O_112_]^8−^ clusters are known to form in ammonium molybdate solutions over the pH range 1.0–1.8 (Falaise *et al.*, 2018 ▸; Himeno *et al.*, 1997 ▸), the only structural report of an ammonium salt of the Mo_36_O_112_]^8−^ cluster is [NH_4_]_4_[Hmorph]_6_[Cu(H_2O_)_2_][Mo_36_O_114_(H_2_O)_14_]·28H_2_O (Gao *et al.*, 2019 ▸). Here, we report the single-crystal structure of (NH_4_)_12_[Mo_36_O_112_(OH)_4_(H_2_O)_10_]·26H_2_O, representing a new polymeric form of the [Mo_36_O_112_]^8−^ isopolymolybdate anion. The scheme shows the asymmetric unit of [Mo_36_O_112_(OH)_4_(H_2_O)_10_]^12−^. The arrows indicate the direction of extension of the polymeric structure. The two hydroxo groups are shown in red and the two MoO_7_ penta­gonal bipyramids are shown in teal.
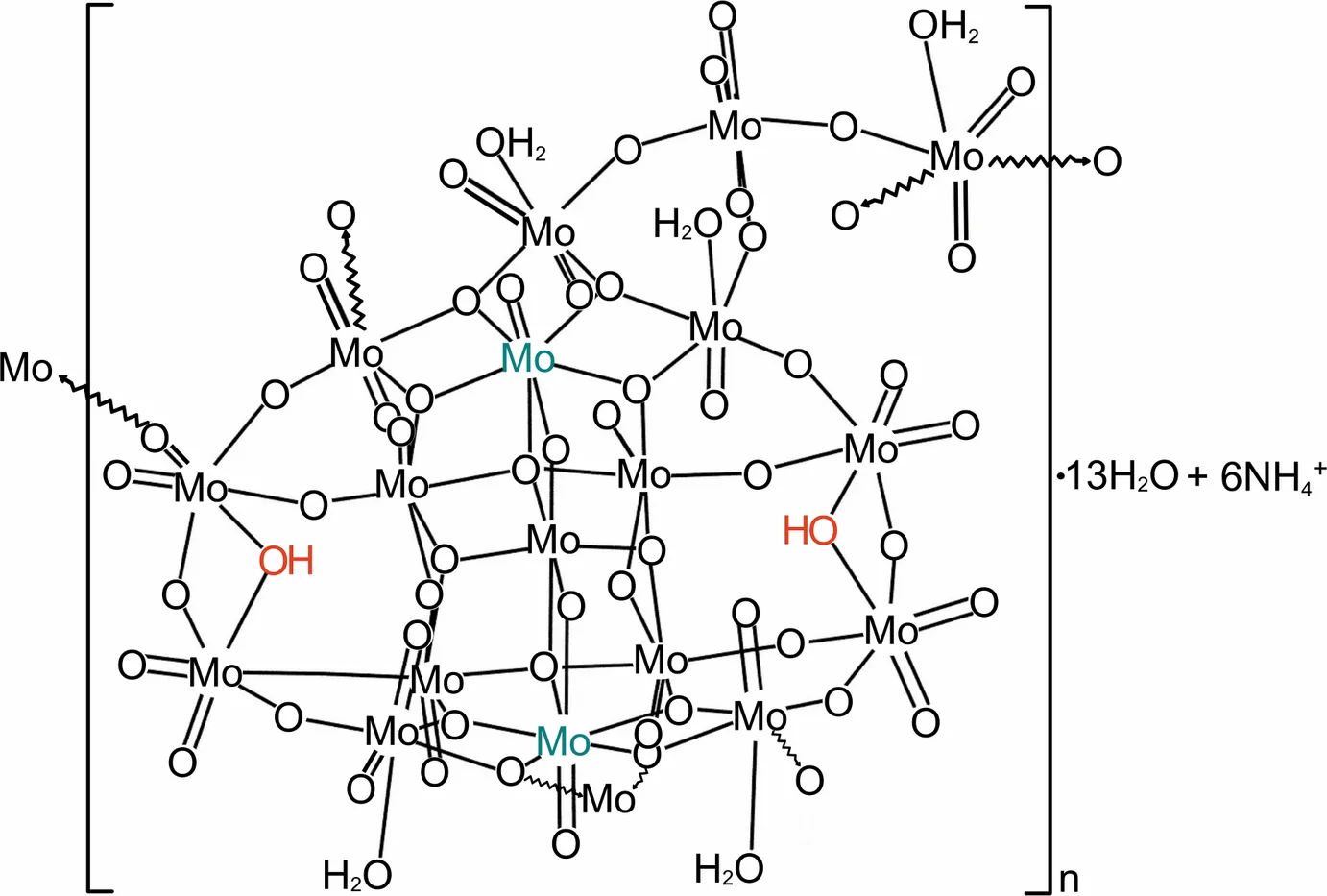


## 2. Structural commentary

**Figure 1:**
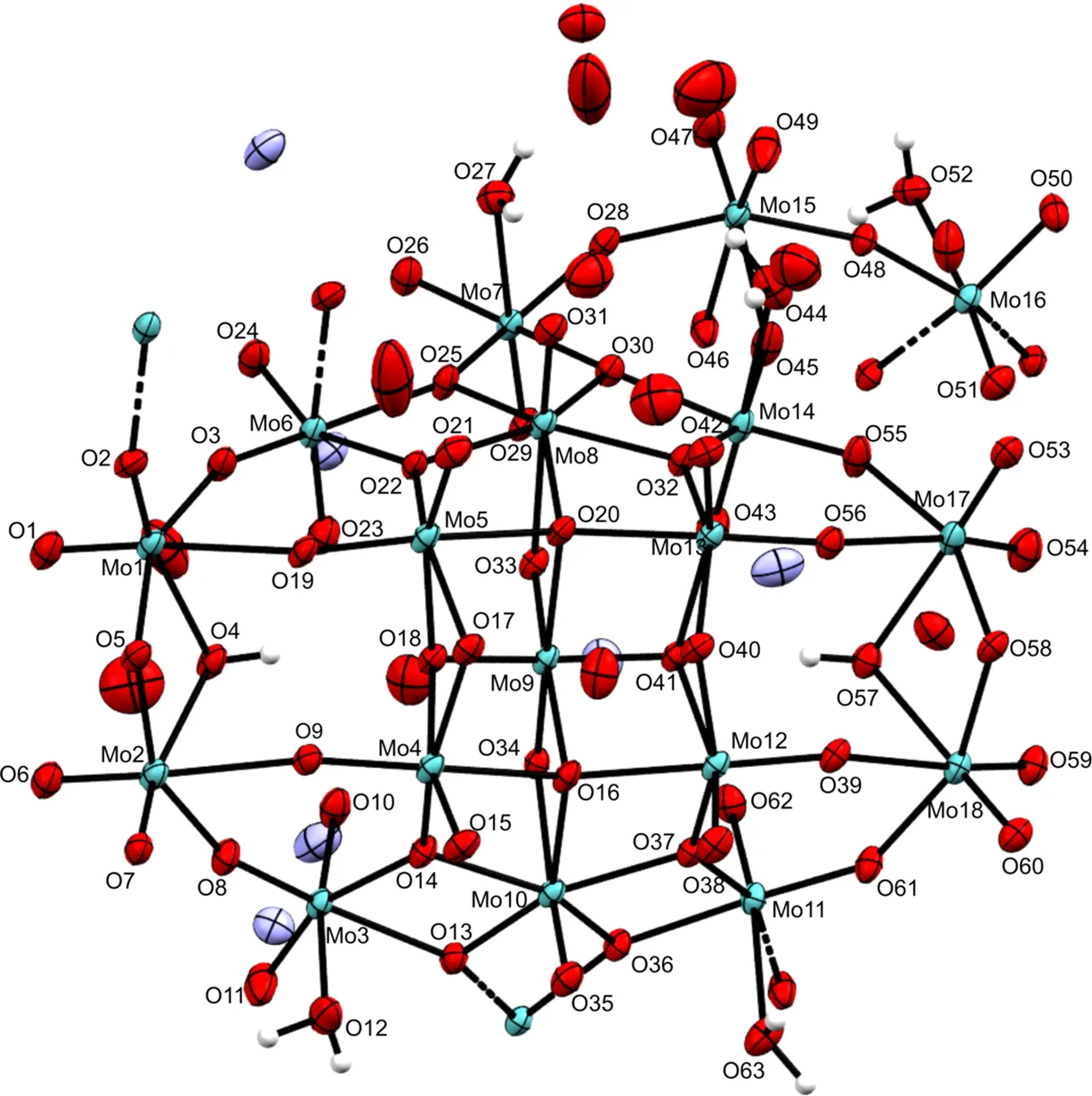
The asymmetric unit of (NH_4_)_12_[Mo_36_O_112_(OH)_4_(H_2_O)_10_]·26H_2_O using 50% probability displacement ellipsoids. Labels and H atoms on the solvent water mol­ecules and counter-ions have been omitted for clarity. The minor positionally disordered atoms were also omitted. The dashed lines indicate extension of the polymeric structure where the symmetry equivalent atoms were not labeled.

Fig. 1 ▸ shows the asymmetric unit of the title structure. The asymmetric unit contains 18 molybdate fragments com­posed primarily of MoO_6_ octa­hedra, along with two MoO_7_ penta­gonal bipyramids (see scheme, shown in teal). Bond-valence analysis (Table 1 ▸) supports Mo^VI^ for all molybdenum centers. The Mo—O bond distances span 1.674 (3)–2.465 (3) Å and were used to support the assignment of hydroxo, oxo, and aqua ligands, following criteria established in related structures (Garin & Costamagna, 1988 ▸; Baizig *et al.*, 2018 ▸; Paulat-Böschen, 1979 ▸; Sukmana *et al.*, 2024 ▸; Atencio *et al.*, 2005 ▸). The distances in the title structure can be grouped into six categories: terminal Mo—O, Mo—OH_2_, bridging Mo—OH—Mo, and three types of bridging oxo ligands in which the O atom is two-, three-, or four-coordinate. The bond-length ranges for each bond type are summarized in Table 2 ▸. Briefly, terminal Mo—O bonds range from 1.674 (3) to 1.720 (3) Å, bridging oxo bonds from 1.730 (3) to 2.364 (3) Å, and Mo—OH_2_ bonds from 2.304 (5) to 2.465 (3) Å, consistent with related polyoxomolybdate structures (Garin & Costamagna, 1988 ▸; Baizig *et al.*, 2018 ▸; Paulat-Böschen, 1979 ▸; Sukmana *et al.*, 2024 ▸; Atencio *et al.*, 2005 ▸).

Two bridging hydroxo groups are assigned in this structure. The Mo1—O4, Mo2—O4, Mo17—O57, and Mo18—O57 bond distances are approximately 2.2 Å (Table 3 ▸). By com­parison with related structures, Mo—O distances near 2.2 Å are consistent with hydroxo ligands, whereas longer distances near 2.4 Å are characteristic of aqua ligands; therefore, O4 and O57 were assigned as hydroxo sites (Sukmana *et al.*, 2024 ▸; Atencio *et al.*, 2005 ▸). This assignment is further supported by the presence of electron density consistent with one H atom for these O atoms in the difference Fourier maps. In contrast, for the structure K_10_[Mo_36_O_110_(OH)_6_(H_2_O)_12_]·38H_2_O, Baizig *et al.* (2018 ▸) assigned atom O25 as hydroxo despite its triply bridging environment and a reported Mo—O distance of 1.970 Å. In the title structure, the corresponding Mo6—O25, Mo7—O25, and Mo8—O25 dis­tances are 2.038 (3), 2.050 (3), and 2.019 (3) Å, respectively. Although these values are com­parable, assignment of O25 as hydroxo appears inconsistent with other closely related Mo_36_ clusters, in which similar bonding environments and bond lengths were not assigned as hydroxo sites, but as oxo (Atencio *et al.*, 2005 ▸; Sukmana *et al.*, 2024 ▸; Paulat-Böschen, 1979 ▸). On this basis, the cluster formulation and charge are assigned as [Mo_36_O_112_(OH)_4_(H_2_O)_10_]^12−^. The molybdenum coordination environments in [Mo_36_O_112_(OH)_4_(H_2_O)_10_]^12−^ are distributed as follows: MoO_6_, 14; MoO_5_OH_2_, 10; MoO_5_OH, 8; and MoO_7_, 4.

**Figure 2:**
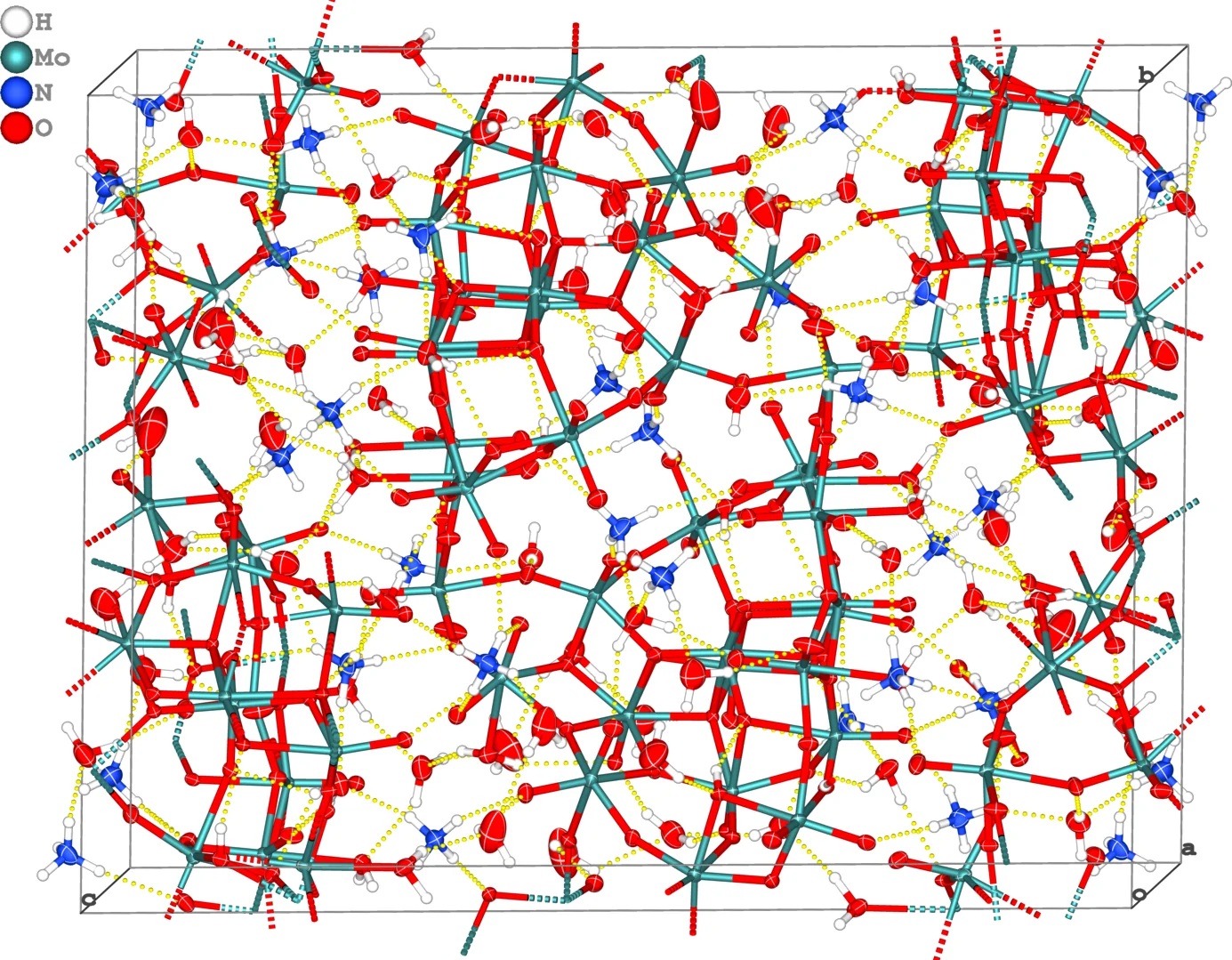
One unit cell of (NH_4_)_12_[Mo_36_O_112_(OH)_4_(H_2_O)_10_]·26H_2_O, illustrating the extensive hy­dro­gen-bonding network (yellow dashes). Each [Mo_36_O_112_(OH)_4_(H_2_O)_10_]^12−^ cluster contains an inter­nal cavity occupied by two ammonium cations and two solvent water mol­ecules, while inter­vening layers of solvent water mol­ecules and ammonium cations form an extended network that connects neighboring clusters.

Charge balance for the [Mo_36_O_112_(OH)_4_(H_2_O)_10_]^12−^ anion requires 12 ammonium cations, giving an overall formulation of (NH_4_)_12_[Mo_36_O_112_(OH)_4_(H_2_O)_10_]·26H_2_O. Distinguishing ammonium cations from solvent water mol­ecules in X-ray diffraction is challenging and the associated H atoms are weakly defined in the electron-density maps. Nevertheless, four ammonium ions (N1*A*–N4*A*) were clearly identified from the observation of four peaks in the difference Fourier maps, consistent with four H atoms, whereas a few water mol­ecules were similarly assigned based on the presence of two corresponding H-atom peaks. Considerable effort was made to assign and position the remaining H atoms using residual positive electron density, together with the local arrangement of hy­dro­gen-bond donors and acceptors. Disorder in the polyoxomolybdate anion further com­plicated the refinement, requiring many water mol­ecules and one ammonium cation to be modeled over two or more sites. Although the resulting model provides the most chemically reasonable inter­pretation of the available crystallographic data, some uncertainty remains in the assignments of the disordered ammonium and water sites, and in the positions of their H atoms. To our knowledge, no previous structural study of a Mo_36_ isopolymolybdate has undertaken such an extensive analysis to distinguish the ammonium cations and water mol­ecules and to assign their H atoms. This detailed treatment reveals that these solvated mol­ecules form an extensive hy­dro­gen-bonding network that creates an inter­vening layer and connects neighboring polyoxomolybdate units within the crystal packing (Fig. 2 ▸).

## 3. Supra­molecular features

**Figure 3:**
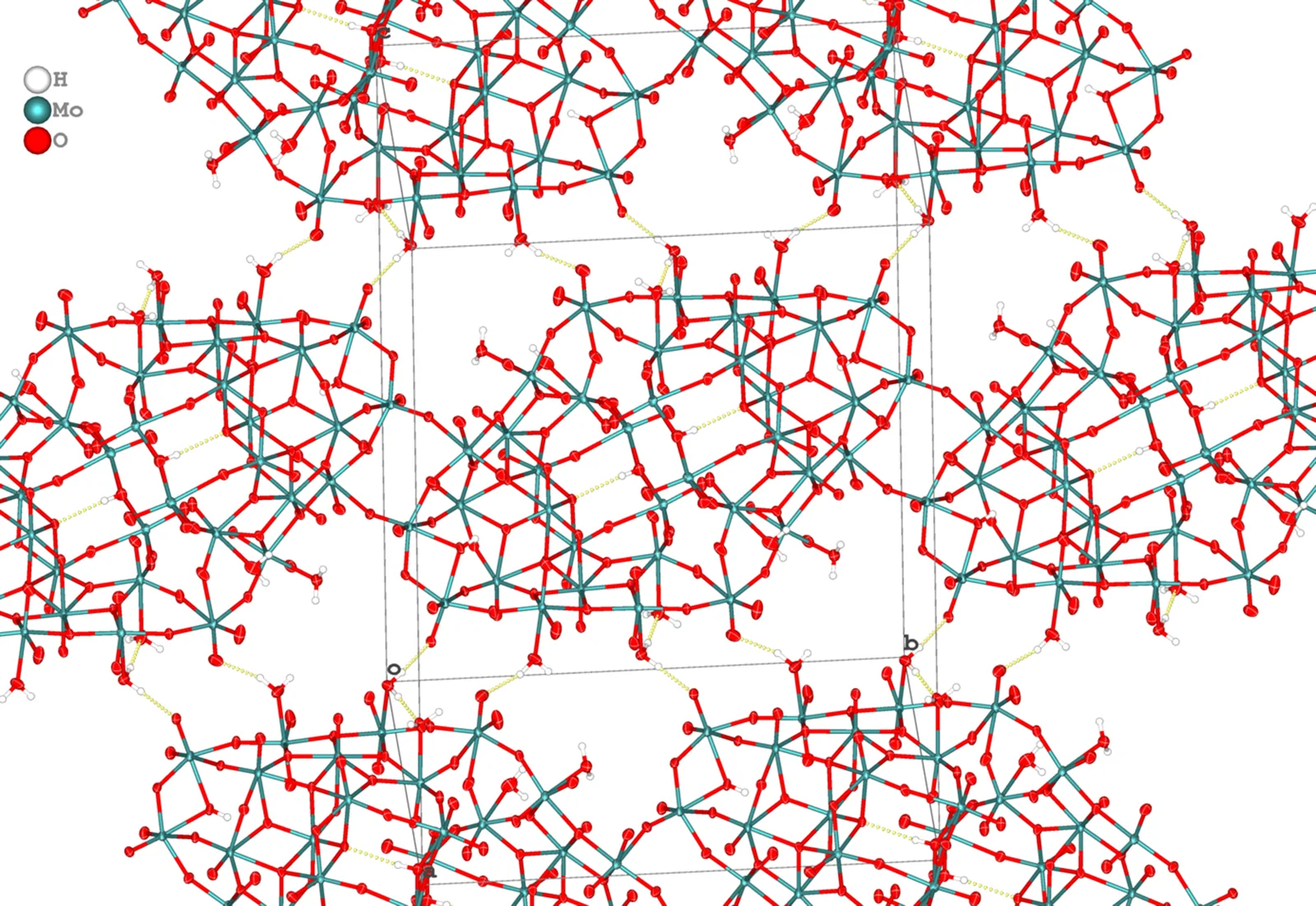
Extended polymeric structure of [Mo_36_O_112_(OH)_4_(H_2_O)_10_]^12−^, showing cage-like units connected by two oxo bridges along the *b* axis, with hy­dro­gen bonds (yellow dashed lines) linking adjacent layers along the *c* axis.

**Figure 4:**
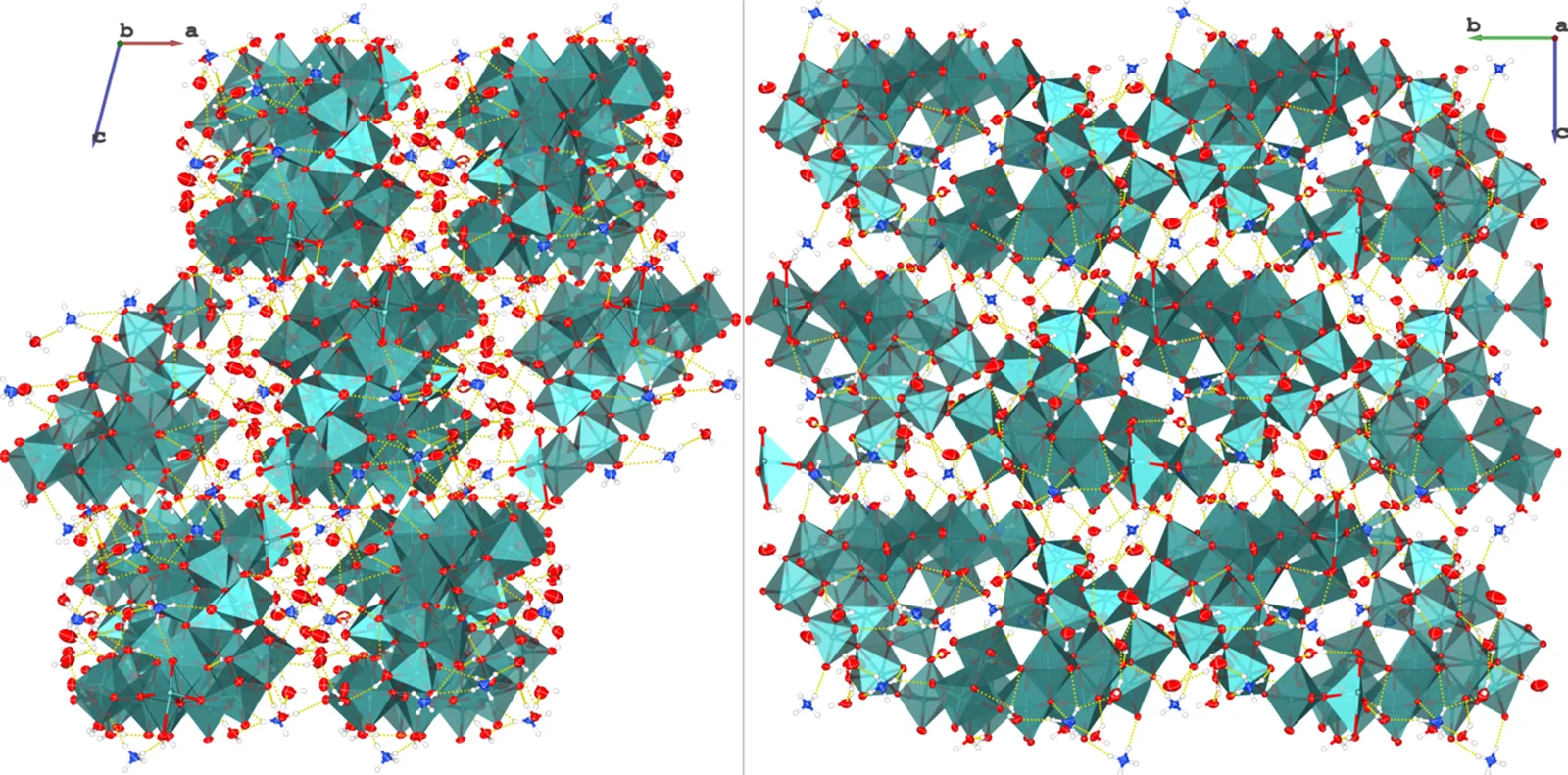
Polyhedral views of (NH_4_)_12_[Mo_36_O_112_(OH)_4_(H_2_O)_10_]·26H_2_O projected along the *b* (left) and *a* (right) axes, illustrating the extensive hy­dro­gen-bonding network surrounding the isopolymolybdate anions and the inter­nal cavity occupied by two water mol­ecules and two ammonium cations. Molybdenum polyhedra are shown in teal, with O atoms in red, N atoms in blue, and H atoms in white.

The supra­molecular structure of the title compound is shown in Fig. 3 ▸ without the solvated mol­ecules or ammonium counter-ions. The structure extends through two oxo bridges along the *b* axis, while hy­dro­gen bonding from the aqua ligands connects the structures along the *c* axis. As shown in the polyhedral representation (Fig. 4 ▸), each cluster exhibits a large cavity that holds two solvated water mol­ecules and two ammonium cations, with an extensive network of hy­dro­gen bonding between solvent water mol­ecules and the remaining ammonium cations. This network of water mol­ecules and ammonium cations links neighboring clusters throughout the crystal structure.

## 4. Database survey

Looking for related structures, we conducted database searches in the Cambridge Structural Database (CSD, accessed July 2026; Groom *et al.*, 2016 ▸). Several single-crystal structural studies have also been published on a variety of related isopolymolybdate species, including ammonium dimolybdate (Armour *et al.*, 1975 ▸), potassium tetra­molybdate (Gatehouse & Leverett, 1971 ▸), and hexa­kis­(*n*-pentyl­ammonium) hepta­molybdate(VI) trihydrate (Román *et al.*, 1988 ▸).

Several molybdate solutions containing isopolymolybdate Mo_36_ clusters paired with inorganic cations K_10_[Mo_36_O_110_(OH)_6_(H_2_O)_12_]·38H_2_O (Baizig *et al.*, 2018 ▸) or organic cations were discussed above (Sukmana *et al.*, 2024 ▸; Garin & Costamagna, 1988 ▸; Eda & Iriki, 2005 ▸; Atencio *et al.*, 2005 ▸), as well as other isopolymolybdate Mo_36_ structures, including Na_6_[HO(CH_2_CH_2_O)_4_H]_3_[Mo_36_O_108_(H_2_O)_14_(OH)_6_{HO(CH_2_CH_2_O)_3_H}_2_]·75H_2_O (Ma *et al.*, 2009 ▸), [HN(CH_2_CH_2_OH)_3_]_2_[H_14_Mo_36_O_116_(OH)_2_(H_2_O)_8_{(HN(CH_2_CH_2_OH)_3_}_2_]·12H_2_O (Priya *et al.*, 2026 ▸), [NH_4_]_4_[Hmorph]_6_[Cu(H_2_O)_2_][Mo_36_O_114_(H_2_O)_14_]·28H_2_O (Gao *et al.*, 2019 ▸), and (TEAH)_6_[Na_2_{Mo_36_O_112_(H_2_O)_14_}]·28H_2_O (deposition No. 292391) (Long *et al.*, 2006 ▸). These isopolymolybdate Mo_36_ clusters share a similar cage-like framework, and several exhibit positional disorder between terminal aqua and oxo ligands (Priya *et al.*, 2026 ▸; Gao *et al.*, 2019 ▸; Sukmana *et al.*, 2024 ▸). These reported structures commonly contain either four water mol­ecules (Eda & Iriki, 2005 ▸; Gao *et al.*, 2019 ▸) or two water mol­ecules and two cations within the central cavity (Baizig *et al.*, 2018 ▸; Sukmana *et al.*, 2024 ▸; Ma *et al.*, 2009 ▸; Long *et al.*, 2006 ▸). The position of these mol­ecules or ions, including small cations such as Na^+^ and K^+^, closely resemble those observed in the title com­pound. However, in most previously reported structures, the solvent water mol­ecules were not modeled explicitly or were treated using a solvent-masking procedure such as SQUEEZE (Spek, 2015 ▸). In some cases, the NH_4_^+^ cations were not distinguished from water mol­ecules (Gao *et al.*, 2019 ▸). Many of these com­pounds also crystallize in the space group *P*2_1_/*n* with unit-cell dimensions similar to those of the title com­pound, with the modest variations likely arising from differences in the counter-ions (Eda & Iriki, 2005 ▸; Baizig *et al.*, 2018 ▸; Atencio *et al.*, 2005 ▸).

The most detailed previous analysis of the hy­dro­gen-bonding network was reported by Priya *et al.* (2026 ▸). In that structure, however, the [Mo_36_] polyoxomolybdate is functionalized with tri­ethano­lamine, producing covalent links between clusters along the [110] direction; no hy­dro­gen bonding involving the aqua ligands along the *c* axis, or solvent-mediated hy­dro­gen bonding between clusters in other directions, was described. By contrast, the title com­pound is modeled in substanti­ally greater detail and reveals a continuous three-dimensional network in which neighboring clusters are linked by hy­dro­gen bonds involving coordinated aqua ligands, solvent water mol­ecules, and ammonium cations.

## 5. Synthesis and crystallization

An ammonium hepta­molybdate solution in 0.05 *N* nitric acid at pH ∼ 1.8 was heated to 343–353 K and then cooled slowly to room tem­per­a­ture. A small amount (∼100 mg) of seed crystals of the ammonium salt of Mo-13 isopolymolybdate was subsequently added (Garin & Costamagna, 1988 ▸; Gumerova & Rompel, 2020 ▸). The seed crystals remained suspended without dissolving in the ammonium hepta­molybdate solution at approximately pH 2. Single crystals of (NH_4_)_12_[Mo_36_O_112_(OH)_4_(H_2_O)_10_]·26H_2_O formed over a period of one month by slow evaporation of the solvent at room tem­per­a­ture.

## 6. Refinement

The crystal chosen for data collection was found to contain two domains (second domain approximately 3%) that were treated as a non-merohedral twin. The H atoms of the water mol­ecules and ammonium cations were located from difference Fourier maps and refined isotropically using a riding model. Crystal data, data collection and structure refinement details are summarized in Table 4 ▸.

## Supplementary Material

Crystal structure: contains datablock(s) I. DOI: 10.1107/S2056989026010388/oo2020sup1.cif

Structure factors: contains datablock(s) I. DOI: 10.1107/S2056989026010388/oo2020Isup2.hkl

CCDC reference: 2593690

Additional supporting information:  crystallographic information; 3D view; checkCIF report

## Figures and Tables

**Table 1:** Bond-valence-sum values for Mo in (NH_4_)_12_[Mo_36_O_112_(OH)_4_(H_2_O)_10_]·26H_2_O

Mo1	6.09	Mo10	5.99
Mo2	6.03	Mo11	5.92
Mo3	6.12	Mo12	6.10
Mo4	6.04	Mo13	6.06
Mo5	6.07	Mo14	6.27
Mo6	6.17	Mo15	6.23
Mo7	6.11	Mo16	5.80
Mo8	6.01	Mo17	6.08
Mo9	5.91	Mo18	6.00

**Table 2:** Bond types and bond distance ranges (Å) of Mo—O in the structure of (NH_4_)_12_[Mo_36_O_112_(OH)_4_(H_2_O)_10_]·26H_2_O

Terminal Mo–O	1.674 (3)–1.720 (3)
Mo—OH_2_	2.304 (5)–2.465 (3)
Bridging Mo—OH—Mo	2.167 (3)–2.235 (4)
Doubly bound oxo	1.730 (3)–2.332 (3)
Triply bound oxo	1.863 (3)–2.279 (3)
Quadruply bound oxo	2.047 (3)–2.364 (3)

**Table 3:** Selected bond lengths (Å)

Mo1—O4	2.167 (3)	Mo17—O57	2.235 (4)
Mo2—O4	2.197 (3)	Mo18—O57	2.198 (3)

**Table 4:** Experimental details

Crystal data	
Chemical formula	(NH_4_)_12_[Mo_36_O_112_(OH)_4_(H_2_O)_10_]·26H_2_O
*M*_r_	6178.94
Crystal system, space group	Monoclinic, *P*2_1_/*n*
Temperature (K)	100
*a*, *b*, *c* (Å)	13.8258 (7), 19.8968 (10), 25.8705 (12)
β (°)	104.611 (2)
*V* (Å^3^)	6886.5 (6)
*Z*	2
Radiation type	Mo *K*α
μ (mm^−1^)	3.29
Crystal size (mm)	0.07 × 0.04 × 0.02

Data collection	
Diffractometer	Bruker D8 Venture
Absorption correction	Multi-scan (*TWINABS*; Bruker, 2012 ▸)
*T*_min_, *T*_max_	0.456, 0.565
No. of measured, independent and observed [*I* > 2σ(*I*)] reflections	22055, 22055, 17440
*R*_int_	0.162
(sin θ/λ)_max_ (Å^−1^)	0.726

Refinement	
*R*[*F*^2^ > 2σ(*F*^2^)], *wR*(*F*^2^), *S*	0.041, 0.105, 1.10
No. of reflections	22055
No. of parameters	1118
No. of restraints	871
H-atom treatment	H atoms treated by a mixture of independent and constrained refinement
Δρ_max_, Δρ_min_ (e Å^−3^)	2.37, −1.32

Computer programs: *SAINT* (Bruker, 2016 ▸), *SHELXT2018* (Sheldrick, 2015*a* ▸), *SHELXL2025* (Sheldrick, 2015*b* ▸), and *OLEX2* (Dolomanov *et al.*, 2009 ▸).

## supplementary crystallographic information

### Dodecaammoniumhexacosahydrate Crystal data

**Table:**

(NH_4_)_12_[Mo_36_O_112_(OH)_4_(H_2_O)_10_]·26H_2_O	*F*(000) = 5872
*M**_r_* = 6178.94	*D*_x_ = 2.980 Mg m^−^^3^
Monoclinic, *P*2_1_/*n*	Mo *K*α radiation, λ = 0.71073 Å
*a* = 13.8258 (7) Å	Cell parameters from 9368 reflections
*b* = 19.8968 (10) Å	θ = 2.6–31.0°
*c* = 25.8705 (12) Å	µ = 3.29 mm^−^^1^
β = 104.611 (2)°	*T* = 100 K
*V* = 6886.5 (6) Å^3^	Plate, colourless
*Z* = 2	0.07 × 0.04 × 0.02 mm

### Dodecaammoniumhexacosahydrate Data collection

**Table:**

Bruker D8 Venture diffractometer	22055 independent reflections
Radiation source: microfocus sealed tube, Incoatec IµS	17440 reflections with *I* > 2σ(*I*)
Multilayer mirror monochromator	*R*_int_ = 0.162
φ and ω scans	θ_max_ = 31.1°, θ_min_ = 1.8°
Absorption correction: multi-scan (TWINABS; Bruker, 2012)	*h* = −20→19
*T*_min_ = 0.456, *T*_max_ = 0.565	*k* = 0→28
22055 measured reflections	*l* = 0→37

### Dodecaammoniumhexacosahydrate Refinement

**Table:**

Refinement on *F*^2^	Primary atom site location: dual
Least-squares matrix: full	Hydrogen site location: difference Fourier map
*R*[*F*^2^ > 2σ(*F*^2^)] = 0.041	H atoms treated by a mixture of independent and constrained refinement
*wR*(*F*^2^) = 0.105	*w* = 1/[σ^2^(*F*_o_^2^) + (0.0556*P*)^2^ + 8.2413*P*] where *P* = (*F*_o_^2^ + 2*F*_c_^2^)/3
*S* = 1.10	(Δ/σ)_max_ = 0.002
22055 reflections	Δρ_max_ = 2.37 e Å^−^^3^
1118 parameters	Δρ_min_ = −1.32 e Å^−^^3^
871 restraints	

### Dodecaammoniumhexacosahydrate Special details

**Table:**

Geometry. All esds (except the esd in the dihedral angle between two l.s. planes) are estimated using the full covariance matrix. The cell esds are taken into account individually in the estimation of esds in distances, angles and torsion angles; correlations between esds in cell parameters are only used when they are defined by crystal symmetry. An approximate (isotropic) treatment of cell esds is used for estimating esds involving l.s. planes.
Refinement. The crystal contained two domains (second domain approx. 3 percent) and were treated as a non-merohedral twin. A multiscan absorption correction was applied to the main domain with TWINABS-2012/1 and final refinement was performed using the HKLF-4 file. Hydrogen atoms on the oxygen and nitrogen atoms were placed based on difference Fourier maps when identifiable, or positioned to support plausible hydrogen-bonding interactions where such assignments could be reasonably determined. These hydrogen atoms were refined using a riding model with Uiso = 1.5Ueq of the parent atom. Water molecules were initially refined with SADI and DFIX restraints but then fixed using AFIX 3. Ammonium ions were restrained with DFIX and SADI restraints. The disordered Mo-O and Mo-H2O sites were modeled using SADI restraints, and the corresponding oxygen atoms were constrained with EXYZ and EADP. Only one disordered Mo pair required EADP; the remaining disordered Mo atoms refined satisfactorily without a displacement-parameter restraint. The hydrogen atoms on the aqua ligands were fixed with AFIX 3 except those on O64 which were found in the difference Fourier maps and DFIX was used for restraint.Data reduction and integration of both domains were performed with *SAINT* in *APEX6* (Bruker, 2016), and a multiscan absorption correction was applied to the main domain with *TWINABS* (Bruker, 2012). The structure was solved by the dual-space method implemented in *SHELXT2018* (Sheldrick, 2015*a*) and refined by full-matrix least-squares methods against *F*^2^ using *SHELXL2025* (Sheldrick, 2015*b*) within *OLEX2* (Dolomanov *et al.*, 2009). All non-H atoms were refined anisotropically.

### Dodecaammoniumhexacosahydrate Fractional atomic coordinates and isotropic or equivalent isotropic displacement parameters (Å^2^)

**Table:**

	*x*	*y*	*z*	*U*_iso_*/*U*_eq_	Occ. (<1)
Mo1	0.36955 (3)	0.99211 (2)	0.55477 (2)	0.01906 (8)	
Mo2	0.27982 (3)	0.93090 (2)	0.64893 (2)	0.01995 (8)	
Mo3	0.24179 (6)	0.74520 (4)	0.65016 (3)	0.01688 (15)	0.829 (4)
Mo4	0.51799 (3)	0.81450 (2)	0.69445 (2)	0.01667 (7)	
Mo5	0.60628 (3)	0.87680 (2)	0.60033 (2)	0.01711 (8)	
Mo6	0.42543 (3)	0.87174 (2)	0.45756 (2)	0.01878 (8)	
Mo7	0.51965 (5)	0.74090 (3)	0.37581 (2)	0.01607 (16)	0.921 (3)
Mo8	0.63580 (3)	0.78213 (2)	0.50201 (2)	0.01651 (7)	
Mo9	0.53069 (3)	0.71084 (2)	0.58988 (2)	0.01525 (7)	
Mo10	0.45470 (3)	0.65576 (2)	0.69371 (2)	0.01572 (7)	
Mo11	0.52071 (3)	0.50216 (2)	0.66643 (2)	0.01675 (7)	
Mo12	0.69803 (3)	0.65227 (2)	0.70375 (2)	0.01574 (7)	
Mo13	0.78559 (3)	0.71442 (2)	0.60971 (2)	0.01624 (7)	
Mo14	0.69995 (4)	0.63101 (2)	0.47252 (2)	0.01854 (12)	0.9627 (15)
Mo15	0.68154 (3)	0.62416 (2)	0.32289 (2)	0.01783 (8)	
Mo16	0.74824 (3)	0.44219 (2)	0.33768 (2)	0.01699 (7)	
Mo17	0.87245 (3)	0.53451 (2)	0.57841 (2)	0.02073 (8)	
Mo18	0.78681 (3)	0.47406 (2)	0.67398 (2)	0.01820 (8)	
Mo1B	0.7418 (8)	0.6427 (5)	0.4658 (4)	0.01854 (12)	0.0373 (15)
Mo3B	0.2510 (4)	0.7509 (2)	0.6729 (3)	0.0367 (13)	0.171 (4)
Mo7B	0.5543 (7)	0.7649 (5)	0.3698 (3)	0.031 (2)	0.079 (3)
O1	0.2535 (3)	1.01949 (18)	0.52319 (13)	0.0273 (7)	
O2	0.4467 (3)	1.06141 (16)	0.55699 (12)	0.0224 (7)	
O3	0.4017 (3)	0.94460 (17)	0.49668 (12)	0.0231 (7)	
O4	0.3126 (3)	0.89653 (16)	0.57445 (13)	0.0225 (7)	
H4	0.352 (4)	0.865 (2)	0.574 (2)	0.034*	
O5	0.3608 (3)	1.00048 (16)	0.62785 (12)	0.0208 (6)	
O6	0.1641 (3)	0.96108 (17)	0.61818 (14)	0.0262 (7)	
O7	0.2980 (2)	0.95710 (16)	0.71448 (13)	0.0216 (6)	
O8	0.2457 (3)	0.83929 (16)	0.65584 (13)	0.0235 (7)	
O9	0.4364 (2)	0.88192 (15)	0.68214 (12)	0.0189 (6)	
O10	0.2484 (3)	0.73522 (18)	0.58621 (13)	0.0270 (7)	0.829 (4)
O11	0.1223 (3)	0.72607 (19)	0.64967 (18)	0.0375 (9)	
O12	0.2732 (4)	0.7587 (3)	0.74134 (18)	0.0325 (13)	0.829 (4)
H12A	0.253953	0.725791	0.758098	0.049*	0.829 (4)
H12B	0.239595	0.790149	0.751012	0.049*	0.829 (4)
O13	0.3039 (2)	0.65444 (15)	0.67721 (12)	0.0187 (6)	
O14	0.4058 (2)	0.74974 (15)	0.68204 (12)	0.0191 (6)	
O15	0.5623 (3)	0.81185 (16)	0.76241 (12)	0.0224 (7)	
O16	0.5705 (2)	0.71530 (15)	0.68422 (11)	0.0179 (6)	
O17	0.6316 (2)	0.85045 (16)	0.67425 (11)	0.0186 (6)	
O18	0.4919 (2)	0.79644 (15)	0.60583 (12)	0.0181 (6)	
O19	0.5166 (3)	0.93821 (15)	0.59712 (12)	0.0207 (6)	
O20	0.6640 (2)	0.78185 (15)	0.58360 (11)	0.0170 (6)	
O21	0.7137 (3)	0.91840 (17)	0.60150 (13)	0.0250 (7)	
O22	0.5541 (2)	0.85444 (15)	0.52358 (12)	0.0184 (6)	
O23	0.3503 (3)	0.81296 (18)	0.47478 (14)	0.0273 (7)	
O24	0.3654 (3)	0.89090 (18)	0.39278 (13)	0.0262 (7)	
O25	0.5223 (2)	0.80485 (16)	0.43829 (12)	0.0201 (6)	
O26	0.4394 (3)	0.79049 (17)	0.33051 (13)	0.0261 (7)	
O27	0.6351 (3)	0.82871 (17)	0.36944 (14)	0.0266 (7)	0.921 (3)
H27A	0.669871	0.822405	0.346485	0.040*	0.921 (3)
H27B	0.676866	0.838481	0.399056	0.040*	0.921 (3)
O28	0.5946 (2)	0.69736 (15)	0.33499 (12)	0.0195 (6)	
O29	0.4454 (3)	0.6796 (2)	0.3897 (2)	0.0222 (10)	0.921 (3)
O30	0.6374 (2)	0.71854 (16)	0.44384 (11)	0.0182 (6)	
O31	0.7272 (2)	0.83340 (16)	0.49436 (12)	0.0206 (6)	
O32	0.7252 (2)	0.70394 (15)	0.53184 (12)	0.0188 (6)	
O33	0.5149 (2)	0.71308 (15)	0.52087 (12)	0.0175 (6)	
O34	0.4368 (2)	0.65955 (15)	0.60241 (11)	0.0157 (6)	
O35	0.4792 (3)	0.65604 (16)	0.76017 (12)	0.0223 (7)	
O36	0.4085 (2)	0.56245 (15)	0.67541 (12)	0.0174 (6)	
O37	0.5748 (2)	0.59750 (15)	0.69220 (12)	0.0172 (6)	
O38	0.7207 (3)	0.66694 (16)	0.77026 (12)	0.0223 (7)	
O39	0.7689 (2)	0.58161 (16)	0.69888 (12)	0.0194 (6)	
O40	0.7768 (2)	0.72082 (15)	0.68227 (11)	0.0181 (6)	
O41	0.6459 (2)	0.65830 (15)	0.61312 (12)	0.0177 (6)	
O42	0.8730 (3)	0.77478 (17)	0.61056 (13)	0.0226 (7)	
O43	0.5987 (3)	0.59300 (17)	0.48569 (14)	0.0278 (7)	0.9627 (15)
O44	0.8432 (3)	0.6925 (2)	0.46011 (15)	0.0343 (9)	0.9627 (15)
H44A	0.826191	0.717794	0.430692	0.051*	0.9627 (15)
H44B	0.863641	0.722568	0.486243	0.051*	0.9627 (15)
O45	0.6984 (3)	0.60485 (19)	0.40815 (13)	0.0295 (8)	
O46	0.5467 (2)	0.56915 (16)	0.32569 (14)	0.0237 (7)	
O47	0.6478 (3)	0.6303 (2)	0.25515 (14)	0.0345 (9)	
O48	0.7357 (2)	0.53530 (15)	0.32831 (12)	0.0192 (6)	
O49	0.7912 (3)	0.6682 (2)	0.33981 (19)	0.0396 (10)	
O50	0.8535 (2)	0.42581 (17)	0.31634 (14)	0.0249 (7)	
O51	0.7850 (3)	0.43585 (18)	0.40523 (13)	0.0251 (7)	
O52	0.6752 (3)	0.45053 (17)	0.24058 (12)	0.0246 (7)	
H52A	0.618974	0.466664	0.240705	0.037*	
H52B	0.710818	0.480548	0.232336	0.037*	
O53	0.9901 (3)	0.55730 (18)	0.57667 (13)	0.0266 (7)	
O54	0.8589 (3)	0.45577 (19)	0.55194 (14)	0.0312 (8)	
O55	0.8011 (3)	0.58256 (18)	0.51436 (13)	0.0273 (8)	
O56	0.8489 (2)	0.63870 (16)	0.61091 (12)	0.0195 (6)	
O57	0.7236 (2)	0.52241 (17)	0.59643 (13)	0.0212 (6)	
H57	0.693 (4)	0.5581 (18)	0.595 (2)	0.031 (16)*	
O58	0.9009 (2)	0.51308 (16)	0.65353 (12)	0.0210 (6)	
O59	0.7828 (3)	0.39838 (17)	0.64301 (13)	0.0245 (7)	
O60	0.8526 (2)	0.45932 (17)	0.73884 (13)	0.0236 (7)	
O61	0.6513 (2)	0.47315 (16)	0.68455 (13)	0.0215 (6)	
O62	0.5000 (3)	0.51230 (17)	0.59960 (13)	0.0240 (7)	
O63	0.5366 (3)	0.5009 (2)	0.76270 (14)	0.0291 (8)	
H63A	0.547 (7)	0.464 (5)	0.785 (4)	0.10 (3)*	
H63B	0.589 (6)	0.523 (4)	0.782 (3)	0.05 (2)*	
O10B	0.2484 (3)	0.73522 (18)	0.58621 (13)	0.0270 (7)	0.171 (4)
H10A	0.299372	0.717873	0.578197	0.040*	0.171 (4)
H10B	0.221768	0.768496	0.568059	0.040*	0.171 (4)
O12B	0.235 (2)	0.7495 (19)	0.7354 (7)	0.0325 (13)	0.171 (4)
O27B	0.6351 (3)	0.82871 (17)	0.36944 (14)	0.0266 (7)	0.079 (3)
O29B	0.429 (4)	0.691 (3)	0.383 (4)	0.0222 (10)	0.079 (3)
H29A	0.407123	0.706997	0.409447	0.033*	0.079 (3)
H29B	0.450994	0.650836	0.394237	0.033*	0.079 (3)
O43B	0.5987 (3)	0.59300 (17)	0.48569 (14)	0.0278 (7)	0.0373 (15)
H43A	0.605615	0.559002	0.505627	0.042*	0.0373 (15)
H43B	0.570351	0.621889	0.501048	0.042*	0.0373 (15)
O44B	0.8432 (3)	0.6925 (2)	0.46011 (15)	0.0343 (9)	0.0373 (15)
O1W	0.2750 (4)	0.6810 (3)	0.4921 (2)	0.0420 (13)	0.829 (4)
H1WA	0.280637	0.693466	0.523891	0.063*	0.829 (4)
H1WB	0.287913	0.715600	0.476132	0.063*	0.829 (4)
O2W	0.273 (2)	0.6840 (14)	0.4635 (12)	0.045 (6)	0.171 (4)
H2WA	0.268272	0.674151	0.493784	0.068*	0.171 (4)
H2WB	0.291806	0.724773	0.463202	0.068*	0.171 (4)
O3W	0.1296 (15)	0.7609 (13)	0.4247 (7)	0.059 (5)	0.251 (11)
H3WA	0.184681	0.743337	0.440615	0.089*	0.251 (11)
H3WB	0.131912	0.800330	0.437114	0.089*	0.251 (11)
O4W	0.1235 (5)	0.8226 (4)	0.3986 (2)	0.058 (2)	0.749 (11)
H4WA	0.176990	0.839625	0.417129	0.087*	0.749 (11)
H4WB	0.090579	0.855452	0.382399	0.087*	0.749 (11)
O5W	0.1301 (4)	0.8579 (3)	0.5034 (2)	0.0633 (15)	
H5WA	0.158124	0.883977	0.528483	0.095*	
H5WB	0.161804	0.821324	0.510177	0.095*	
O6W	0.8291 (5)	1.0515 (4)	0.6473 (2)	0.083 (3)	0.915 (13)
H6WA	0.775726	1.056786	0.656991	0.124*	0.915 (13)
H6WB	0.821837	1.014449	0.630763	0.124*	0.915 (13)
O7W	0.7773 (4)	0.8369 (3)	0.31325 (18)	0.0375 (11)	0.829 (4)
H7WA	0.790144	0.877480	0.308193	0.056*	0.829 (4)
H7WB	0.828637	0.822497	0.335665	0.056*	0.829 (4)
O8W	0.4655 (17)	0.7536 (15)	0.8474 (8)	0.107 (12)	0.294 (13)
H8WA	0.450052	0.714440	0.835590	0.161*	0.294 (13)
H8WB	0.414342	0.768161	0.855897	0.161*	0.294 (13)
O9W	0.778 (6)	1.031 (4)	0.677 (2)	0.071 (11)	0.085 (13)
H9WA	0.724590	1.051932	0.676458	0.107*	0.085 (13)
H9WB	0.759130	0.991179	0.667857	0.107*	0.085 (13)
O10W	0.8141 (4)	0.8393 (3)	0.7574 (2)	0.0341 (11)	0.829 (4)
H10C	0.843687	0.874134	0.771944	0.051*	0.829 (4)
H10D	0.771734	0.862270	0.735237	0.051*	0.829 (4)
O11W	0.848 (2)	0.8556 (15)	0.7297 (15)	0.059 (7)	0.171 (4)
H11A	0.809516	0.847867	0.699365	0.089*	0.171 (4)
H11B	0.833655	0.883745	0.751101	0.089*	0.171 (4)
O12W	0.9903 (4)	0.8978 (2)	0.66757 (19)	0.0519 (12)	
H12C	0.952233	0.911391	0.638555	0.078*	
H12D	1.048645	0.901175	0.663176	0.078*	
O13W	0.930 (3)	1.0483 (19)	0.5806 (10)	0.082 (8)	0.203 (11)
H13A	0.990396	1.053162	0.580482	0.122*	0.203 (11)
H13B	0.915552	1.008488	0.570059	0.122*	0.203 (11)
O14W	0.9002 (10)	0.9731 (10)	0.5740 (5)	0.058 (4)	0.359 (12)
H14A	0.896544	1.015398	0.572702	0.087*	0.359 (12)
H14B	0.855828	0.961112	0.589374	0.087*	0.359 (12)
O15W	0.9090 (7)	0.9123 (6)	0.5590 (4)	0.043 (3)	0.438 (12)
H15A	0.864113	0.924283	0.573940	0.065*	0.438 (12)
H15B	0.878665	0.888564	0.532754	0.065*	0.438 (12)
O16W	0.9408 (8)	0.9330 (6)	0.4564 (4)	0.111 (5)	0.706 (13)
H16A	0.878640	0.926611	0.445395	0.167*	0.706 (13)
H16B	0.948347	0.953646	0.485635	0.167*	0.706 (13)
O17W	0.9193 (7)	0.8077 (6)	0.4015 (3)	0.085 (4)	0.708 (14)
H17A	0.966796	0.827623	0.392704	0.127*	0.708 (14)
H17B	0.919162	0.768533	0.388862	0.127*	0.708 (14)
O18W	0.8419 (17)	0.8054 (13)	0.4136 (9)	0.082 (7)	0.292 (14)
H18A	0.861097	0.770427	0.431788	0.123*	0.292 (14)
H18B	0.787296	0.794513	0.392181	0.123*	0.292 (14)
O19W	0.9957 (4)	0.7671 (3)	0.5340 (2)	0.0517 (15)	0.829 (4)
H19A	1.009176	0.763841	0.567605	0.078*	0.829 (4)
H19B	0.978555	0.807635	0.527493	0.078*	0.829 (4)
O20W	0.076 (2)	0.6780 (17)	0.4469 (11)	0.057 (6)	0.171 (4)
H20A	0.040974	0.701702	0.422034	0.086*	0.171 (4)
H20B	0.071628	0.638551	0.434485	0.086*	0.171 (4)
O21W	0.9267 (3)	0.5746 (2)	0.42193 (16)	0.0381 (9)	
H21A	0.866080	0.577345	0.420221	0.057*	
H21B	0.945848	0.534469	0.426099	0.057*	
O22W	0.6922 (18)	0.8468 (13)	0.2780 (9)	0.0375 (11)	0.171 (4)
H22A	0.696332	0.889024	0.276050	0.056*	0.171 (4)
H22B	0.677563	0.839537	0.307223	0.056*	0.171 (4)
O23W	0.9928 (3)	0.6286 (2)	0.72156 (14)	0.0302 (8)	
H23A	1.042312	0.609915	0.714069	0.045*	
H23B	0.946014	0.614229	0.696382	0.045*	
N1A	0.4599 (4)	0.9416 (2)	0.31106 (17)	0.0321 (10)	
H1AA	0.423 (3)	0.9742 (17)	0.2933 (17)	0.048*	
H1AB	0.426 (3)	0.921 (2)	0.3307 (17)	0.048*	
H1AC	0.476 (4)	0.913 (2)	0.2883 (16)	0.048*	
H1AD	0.513 (2)	0.962 (2)	0.3309 (16)	0.048*	
N2A	0.2428 (3)	0.7391 (2)	0.35001 (18)	0.0303 (9)	
H2AA	0.209 (3)	0.754 (3)	0.3724 (17)	0.046*	
H2AB	0.278 (3)	0.7040 (16)	0.364 (2)	0.046*	
H2AC	0.279 (3)	0.769 (2)	0.339 (2)	0.046*	
H2AD	0.201 (3)	0.724 (2)	0.3208 (11)	0.046*	
N3A	0.4461 (3)	0.9196 (2)	0.80953 (17)	0.0273 (9)	
H3AA	0.487 (3)	0.9534 (15)	0.8170 (18)	0.041*	
H3AB	0.423 (4)	0.910 (3)	0.8368 (14)	0.041*	
H3AC	0.396 (3)	0.933 (2)	0.7833 (15)	0.041*	
H3AD	0.476 (3)	0.8841 (15)	0.801 (2)	0.041*	
N4A	0.9787 (4)	0.7720 (3)	0.72729 (17)	0.0350 (11)	
H4AA	0.9189 (18)	0.781 (3)	0.7317 (19)	0.053*	
H4AB	0.980 (4)	0.7282 (7)	0.723 (2)	0.053*	
H4AC	1.023 (3)	0.781 (4)	0.7575 (14)	0.053*	
H4AD	0.986 (4)	0.793 (2)	0.6988 (15)	0.053*	
N5A	0.3885 (3)	0.5661 (3)	0.48176 (18)	0.0312 (10)	
H5AA	0.356 (3)	0.539 (2)	0.4571 (17)	0.047*	
H5AB	0.395 (4)	0.546 (3)	0.5124 (14)	0.047*	
H5AC	0.355 (4)	0.6034 (17)	0.481 (2)	0.047*	
H5AD	0.445 (2)	0.577 (3)	0.4759 (18)	0.047*	
N6A	1.025 (2)	0.6847 (16)	0.5495 (13)	0.042 (6)	0.171 (4)
H6AA	1.022799	0.644221	0.557275	0.063*	0.171 (4)
H6AB	0.968632	0.688791	0.536842	0.063*	0.171 (4)
H6AC	1.046264	0.691480	0.517998	0.063*	0.171 (4)
H6AD	1.055174	0.710080	0.568826	0.063*	0.171 (4)
N7A	0.0785 (5)	0.6435 (3)	0.5079 (2)	0.0425 (15)	0.829 (4)
H7AA	0.035 (4)	0.626 (3)	0.4808 (17)	0.064*	0.829 (4)
H7AB	0.090 (5)	0.613 (3)	0.533 (2)	0.064*	0.829 (4)
H7AC	0.134 (3)	0.653 (4)	0.498 (3)	0.064*	0.829 (4)
H7AD	0.053 (5)	0.680 (3)	0.518 (3)	0.064*	0.829 (4)

### Dodecaammoniumhexacosahydrate Atomic displacement parameters (Å^2^)

**Table:**

	*U*^11^	*U*^22^	*U*^33^	*U*^12^	*U*^13^	*U*^23^
Mo1	0.0297 (2)	0.01254 (17)	0.01502 (16)	0.00433 (14)	0.00576 (14)	0.00103 (12)
Mo2	0.0297 (2)	0.01270 (17)	0.01794 (16)	0.00371 (14)	0.00684 (15)	−0.00001 (13)
Mo3	0.0218 (3)	0.0120 (2)	0.0161 (3)	0.00197 (17)	0.0035 (2)	−0.0001 (2)
Mo4	0.02492 (18)	0.01068 (16)	0.01328 (15)	0.00039 (13)	0.00274 (13)	−0.00071 (12)
Mo5	0.02615 (19)	0.01100 (16)	0.01317 (15)	0.00098 (13)	0.00312 (13)	−0.00026 (12)
Mo6	0.02592 (19)	0.01528 (17)	0.01385 (15)	0.00516 (14)	0.00264 (14)	−0.00010 (13)
Mo7	0.0221 (3)	0.0124 (3)	0.01233 (19)	0.00175 (18)	0.00166 (16)	−0.00043 (15)
Mo8	0.02346 (18)	0.01204 (16)	0.01270 (15)	0.00206 (13)	0.00212 (13)	−0.00016 (12)
Mo9	0.02092 (17)	0.01058 (16)	0.01263 (14)	0.00144 (13)	0.00120 (13)	−0.00012 (12)
Mo10	0.02161 (17)	0.01098 (16)	0.01376 (15)	0.00047 (13)	0.00294 (13)	−0.00018 (12)
Mo11	0.01894 (17)	0.01162 (16)	0.01873 (16)	−0.00012 (13)	0.00295 (13)	−0.00171 (13)
Mo12	0.02005 (17)	0.01186 (16)	0.01332 (15)	−0.00047 (13)	0.00048 (13)	0.00019 (12)
Mo13	0.02118 (17)	0.01283 (16)	0.01298 (15)	0.00002 (13)	0.00110 (13)	0.00057 (12)
Mo14	0.0253 (3)	0.01358 (19)	0.01422 (17)	0.00461 (16)	0.00026 (15)	−0.00127 (13)
Mo15	0.02212 (18)	0.01303 (17)	0.01864 (16)	0.00114 (13)	0.00568 (14)	0.00201 (13)
Mo16	0.01993 (17)	0.01279 (17)	0.01750 (16)	0.00103 (13)	0.00332 (13)	0.00047 (13)
Mo17	0.02499 (19)	0.01863 (19)	0.01681 (16)	0.00629 (15)	0.00201 (14)	0.00154 (14)
Mo18	0.01927 (17)	0.01371 (17)	0.01985 (17)	0.00171 (13)	0.00165 (14)	0.00205 (13)
Mo1B	0.0253 (3)	0.01358 (19)	0.01422 (17)	0.00461 (16)	0.00026 (15)	−0.00127 (13)
Mo3B	0.0315 (18)	0.0164 (15)	0.065 (4)	0.0051 (12)	0.017 (2)	0.010 (2)
Mo7B	0.040 (5)	0.029 (4)	0.022 (3)	0.010 (4)	0.007 (3)	0.003 (3)
O1	0.040 (2)	0.0191 (17)	0.0211 (16)	0.0071 (15)	0.0048 (14)	0.0008 (13)
O2	0.0350 (18)	0.0155 (15)	0.0178 (14)	0.0001 (13)	0.0089 (13)	0.0018 (12)
O3	0.0343 (18)	0.0188 (16)	0.0161 (14)	0.0088 (14)	0.0058 (13)	0.0014 (12)
O4	0.0363 (19)	0.0115 (15)	0.0197 (15)	0.0047 (13)	0.0069 (14)	−0.0010 (12)
O5	0.0340 (18)	0.0137 (15)	0.0155 (14)	0.0032 (13)	0.0077 (13)	−0.0007 (11)
O6	0.0326 (18)	0.0196 (17)	0.0256 (16)	0.0054 (14)	0.0056 (14)	0.0023 (13)
O7	0.0275 (17)	0.0155 (15)	0.0226 (15)	0.0048 (12)	0.0079 (13)	0.0022 (12)
O8	0.0289 (17)	0.0137 (15)	0.0273 (16)	0.0040 (13)	0.0060 (14)	0.0001 (12)
O9	0.0276 (16)	0.0118 (14)	0.0182 (14)	0.0009 (12)	0.0076 (12)	−0.0016 (11)
O10	0.0334 (19)	0.0250 (18)	0.0189 (15)	0.0074 (15)	−0.0003 (14)	−0.0008 (13)
O11	0.0307 (19)	0.0200 (18)	0.063 (3)	0.0034 (15)	0.0146 (19)	0.0096 (18)
O12	0.046 (4)	0.028 (3)	0.027 (2)	0.013 (3)	0.015 (2)	−0.0003 (18)
O13	0.0227 (15)	0.0127 (14)	0.0211 (14)	0.0019 (12)	0.0061 (12)	−0.0003 (11)
O14	0.0240 (15)	0.0116 (14)	0.0199 (14)	0.0006 (12)	0.0024 (12)	0.0005 (11)
O15	0.0326 (18)	0.0172 (16)	0.0153 (14)	−0.0027 (13)	0.0020 (13)	−0.0001 (12)
O16	0.0266 (16)	0.0112 (14)	0.0139 (13)	0.0025 (12)	0.0014 (12)	−0.0003 (10)
O17	0.0243 (16)	0.0175 (15)	0.0115 (13)	0.0000 (12)	−0.0004 (11)	−0.0004 (11)
O18	0.0244 (15)	0.0141 (15)	0.0147 (13)	0.0011 (12)	0.0026 (12)	0.0011 (11)
O19	0.0344 (18)	0.0109 (14)	0.0167 (14)	0.0043 (12)	0.0061 (13)	0.0011 (11)
O20	0.0250 (15)	0.0121 (14)	0.0123 (13)	0.0009 (11)	0.0017 (11)	0.0012 (10)
O21	0.0361 (19)	0.0175 (16)	0.0206 (15)	−0.0036 (14)	0.0056 (14)	−0.0006 (12)
O22	0.0274 (16)	0.0142 (15)	0.0143 (13)	0.0067 (12)	0.0062 (12)	0.0022 (11)
O23	0.0322 (18)	0.0206 (17)	0.0279 (17)	0.0041 (14)	0.0054 (14)	0.0009 (14)
O24	0.0355 (19)	0.0252 (18)	0.0164 (14)	0.0101 (15)	0.0041 (13)	0.0005 (13)
O25	0.0287 (17)	0.0151 (15)	0.0140 (13)	0.0065 (12)	0.0007 (12)	−0.0011 (11)
O26	0.0329 (18)	0.0234 (18)	0.0200 (15)	0.0081 (14)	0.0030 (14)	−0.0011 (13)
O27	0.0344 (19)	0.0214 (17)	0.0252 (16)	−0.0018 (14)	0.0098 (14)	−0.0013 (13)
O28	0.0280 (16)	0.0129 (14)	0.0154 (13)	0.0011 (12)	0.0016 (12)	0.0010 (11)
O29	0.030 (2)	0.015 (2)	0.020 (2)	0.0039 (17)	0.0022 (18)	0.0042 (15)
O30	0.0252 (16)	0.0147 (15)	0.0132 (13)	0.0020 (12)	0.0021 (12)	−0.0018 (11)
O31	0.0265 (16)	0.0179 (15)	0.0155 (14)	−0.0003 (13)	0.0018 (12)	−0.0007 (12)
O32	0.0266 (16)	0.0142 (15)	0.0142 (13)	0.0047 (12)	0.0023 (12)	0.0004 (11)
O33	0.0211 (15)	0.0131 (14)	0.0157 (13)	−0.0014 (11)	−0.0001 (11)	−0.0005 (11)
O34	0.0185 (14)	0.0117 (14)	0.0158 (13)	0.0008 (11)	0.0020 (11)	−0.0018 (10)
O35	0.0314 (18)	0.0157 (16)	0.0182 (14)	0.0005 (13)	0.0034 (13)	−0.0009 (12)
O36	0.0220 (15)	0.0102 (14)	0.0194 (14)	−0.0008 (11)	0.0041 (12)	−0.0025 (11)
O37	0.0194 (14)	0.0139 (14)	0.0173 (13)	0.0005 (11)	0.0028 (11)	0.0004 (11)
O38	0.0294 (17)	0.0180 (16)	0.0155 (14)	−0.0020 (13)	−0.0020 (12)	−0.0029 (12)
O39	0.0214 (15)	0.0151 (15)	0.0182 (14)	−0.0004 (12)	−0.0013 (12)	0.0031 (11)
O40	0.0260 (16)	0.0138 (14)	0.0131 (13)	−0.0029 (12)	0.0022 (12)	−0.0005 (11)
O41	0.0250 (16)	0.0124 (14)	0.0145 (13)	0.0014 (12)	0.0030 (12)	−0.0004 (11)
O42	0.0256 (16)	0.0214 (17)	0.0208 (15)	−0.0021 (13)	0.0056 (13)	0.0029 (12)
O43	0.0319 (18)	0.0153 (16)	0.0341 (18)	−0.0013 (14)	0.0044 (15)	−0.0011 (14)
O44	0.0282 (19)	0.045 (2)	0.0278 (18)	0.0046 (17)	0.0034 (15)	−0.0083 (17)
O45	0.042 (2)	0.0254 (18)	0.0160 (15)	0.0133 (16)	−0.0013 (14)	−0.0039 (13)
O46	0.0213 (16)	0.0135 (15)	0.0371 (18)	0.0010 (12)	0.0084 (14)	−0.0014 (13)
O47	0.051 (2)	0.033 (2)	0.0203 (16)	0.0189 (18)	0.0104 (16)	0.0060 (15)
O48	0.0227 (15)	0.0123 (14)	0.0227 (15)	0.0014 (12)	0.0061 (12)	0.0003 (12)
O49	0.0262 (19)	0.0221 (19)	0.069 (3)	0.0002 (15)	0.0100 (19)	0.0097 (19)
O50	0.0208 (16)	0.0186 (16)	0.0361 (18)	0.0027 (13)	0.0085 (14)	−0.0020 (14)
O51	0.0282 (17)	0.0248 (18)	0.0195 (15)	0.0003 (14)	0.0008 (13)	0.0026 (13)
O52	0.0315 (18)	0.0224 (17)	0.0223 (15)	−0.0037 (14)	0.0112 (14)	0.0000 (13)
O53	0.0284 (18)	0.0286 (19)	0.0231 (16)	0.0073 (14)	0.0071 (14)	0.0085 (14)
O54	0.041 (2)	0.0238 (18)	0.0267 (17)	0.0059 (16)	0.0041 (16)	−0.0030 (14)
O55	0.0350 (19)	0.0271 (19)	0.0164 (14)	0.0142 (15)	0.0001 (13)	0.0000 (13)
O56	0.0227 (15)	0.0182 (15)	0.0160 (14)	0.0026 (12)	0.0020 (12)	0.0005 (11)
O57	0.0236 (16)	0.0173 (16)	0.0206 (15)	0.0011 (13)	0.0017 (13)	−0.0011 (12)
O58	0.0247 (16)	0.0190 (16)	0.0168 (14)	0.0009 (12)	0.0008 (12)	0.0046 (12)
O59	0.0266 (17)	0.0157 (16)	0.0288 (17)	0.0014 (13)	0.0025 (14)	−0.0005 (13)
O60	0.0246 (16)	0.0203 (17)	0.0236 (16)	−0.0011 (13)	0.0017 (13)	0.0042 (13)
O61	0.0230 (16)	0.0114 (14)	0.0280 (16)	0.0005 (12)	0.0029 (13)	0.0005 (12)
O62	0.0247 (16)	0.0240 (18)	0.0216 (15)	0.0016 (13)	0.0029 (13)	−0.0071 (13)
O63	0.034 (2)	0.0215 (18)	0.0264 (17)	−0.0030 (15)	−0.0016 (15)	0.0053 (14)
O10B	0.0334 (19)	0.0250 (18)	0.0189 (15)	0.0074 (15)	−0.0003 (14)	−0.0008 (13)
O12B	0.046 (4)	0.028 (3)	0.027 (2)	0.013 (3)	0.015 (2)	−0.0003 (18)
O27B	0.0344 (19)	0.0214 (17)	0.0252 (16)	−0.0018 (14)	0.0098 (14)	−0.0013 (13)
O29B	0.030 (2)	0.015 (2)	0.020 (2)	0.0039 (17)	0.0022 (18)	0.0042 (15)
O43B	0.0319 (18)	0.0153 (16)	0.0341 (18)	−0.0013 (14)	0.0044 (15)	−0.0011 (14)
O44B	0.0282 (19)	0.045 (2)	0.0278 (18)	0.0046 (17)	0.0034 (15)	−0.0083 (17)
O1W	0.051 (3)	0.041 (3)	0.033 (3)	0.003 (2)	0.007 (3)	−0.012 (3)
O2W	0.071 (14)	0.031 (11)	0.039 (12)	0.003 (11)	0.023 (12)	−0.010 (11)
O3W	0.054 (10)	0.092 (12)	0.036 (8)	0.012 (9)	0.019 (7)	0.000 (9)
O4W	0.036 (3)	0.096 (6)	0.039 (3)	0.020 (3)	0.001 (3)	−0.008 (3)
O5W	0.069 (4)	0.063 (4)	0.053 (3)	−0.020 (3)	0.004 (3)	−0.017 (3)
O6W	0.084 (5)	0.107 (6)	0.043 (3)	0.060 (4)	−0.008 (3)	−0.008 (3)
O7W	0.042 (3)	0.043 (3)	0.028 (2)	−0.001 (2)	0.011 (2)	0.002 (2)
O8W	0.089 (17)	0.18 (3)	0.053 (12)	0.087 (18)	0.016 (11)	−0.010 (14)
O9W	0.084 (19)	0.088 (19)	0.033 (17)	0.051 (18)	−0.001 (17)	−0.010 (17)
O10W	0.037 (3)	0.027 (3)	0.032 (2)	0.002 (2)	−0.004 (2)	−0.011 (2)
O11W	0.044 (13)	0.042 (13)	0.073 (15)	−0.011 (11)	−0.020 (12)	−0.021 (12)
O12W	0.056 (3)	0.044 (3)	0.054 (3)	−0.008 (2)	0.012 (2)	−0.005 (2)
O13W	0.105 (16)	0.103 (17)	0.034 (11)	0.034 (15)	0.012 (12)	0.021 (12)
O14W	0.038 (6)	0.107 (11)	0.024 (5)	0.003 (7)	−0.003 (5)	0.019 (7)
O15W	0.038 (5)	0.051 (7)	0.038 (5)	0.000 (5)	0.003 (4)	0.010 (5)
O16W	0.131 (9)	0.147 (11)	0.063 (6)	0.104 (8)	0.039 (6)	0.036 (6)
O17W	0.065 (6)	0.115 (8)	0.067 (6)	0.008 (5)	0.002 (4)	0.038 (5)
O18W	0.057 (11)	0.115 (15)	0.082 (12)	0.035 (11)	0.032 (9)	0.021 (11)
O19W	0.054 (3)	0.059 (4)	0.045 (3)	0.003 (3)	0.018 (3)	−0.002 (3)
O20W	0.058 (12)	0.072 (13)	0.048 (11)	−0.004 (11)	0.026 (10)	0.004 (10)
O21W	0.035 (2)	0.036 (2)	0.038 (2)	0.0163 (17)	−0.0004 (17)	−0.0036 (17)
O22W	0.042 (3)	0.043 (3)	0.028 (2)	−0.001 (2)	0.011 (2)	0.002 (2)
O23W	0.0257 (18)	0.041 (2)	0.0234 (16)	0.0012 (16)	0.0052 (14)	−0.0062 (15)
N1A	0.048 (3)	0.024 (2)	0.0218 (19)	−0.001 (2)	0.0034 (19)	0.0019 (16)
N2A	0.033 (2)	0.025 (2)	0.031 (2)	−0.0015 (18)	0.0052 (18)	0.0060 (18)
N3A	0.025 (2)	0.028 (2)	0.028 (2)	−0.0074 (17)	0.0046 (17)	−0.0052 (17)
N4A	0.037 (3)	0.043 (3)	0.0196 (19)	−0.016 (2)	−0.0038 (18)	0.0007 (19)
N5A	0.031 (2)	0.030 (2)	0.029 (2)	−0.0033 (18)	0.0014 (18)	−0.0100 (18)
N6A	0.026 (11)	0.042 (13)	0.051 (13)	−0.007 (10)	−0.003 (11)	0.008 (12)
N7A	0.061 (4)	0.031 (3)	0.036 (3)	−0.003 (3)	0.013 (3)	0.005 (2)

### Dodecaammoniumhexacosahydrate Geometric parameters (Å, º)

**Table:**

Mo1—O1	1.696 (4)	Mo1B—O30	2.065 (11)
Mo1—O2	1.735 (3)	Mo1B—O32	2.157 (11)
Mo1—O3	1.920 (3)	Mo1B—O45	1.643 (11)
Mo1—O4	2.167 (3)	Mo1B—O55	1.779 (11)
Mo1—O5	1.932 (3)	Mo1B—O43B	2.381 (10)
Mo1—O19	2.316 (3)	Mo1B—O44B	1.753 (10)
Mo2—O4	2.197 (3)	Mo3B—O8	1.810 (5)
Mo2—O5	1.944 (3)	Mo3B—O11	1.798 (7)
Mo2—O6	1.706 (3)	Mo3B—O13	2.046 (5)
Mo2—O7	1.731 (3)	Mo3B—O14	2.093 (5)
Mo2—O8	1.902 (3)	Mo3B—O10B	2.254 (8)
Mo2—O9	2.332 (3)	Mo3B—O12B	1.687 (14)
Mo3—O8	1.878 (3)	Mo7B—O25	2.089 (7)
Mo3—O10	1.692 (3)	Mo7B—O26	1.732 (8)
Mo3—O11	1.693 (4)	Mo7B—O28	1.782 (7)
Mo3—O12	2.304 (5)	Mo7B—O30	2.175 (7)
Mo3—O13	2.046 (3)	Mo7B—O27B	1.693 (12)
Mo3—O14	2.211 (3)	Mo7B—O29B	2.375 (16)
Mo4—O9	1.730 (3)	O4—H4	0.83 (2)
Mo4—O14	1.979 (3)	O12—H12A	0.8636
Mo4—O15	1.710 (3)	O12—H12B	0.8536
Mo4—O16	2.142 (3)	O27—H27A	0.8622
Mo4—O17	1.915 (3)	O27—H27B	0.8574
Mo4—O18	2.258 (3)	O44—H44A	0.8927
Mo5—O17	1.928 (3)	O44—H44B	0.8931
Mo5—O18	2.279 (3)	O52—H52A	0.8423
Mo5—O19	1.727 (3)	O52—H52B	0.8350
Mo5—O20	2.137 (3)	O57—H57	0.82 (2)
Mo5—O21	1.694 (4)	O63—H63A	0.92 (11)
Mo5—O22	1.985 (3)	O63—H63B	0.88 (8)
Mo6—O2^i^	2.318 (3)	O10B—H10A	0.8564
Mo6—O3	1.844 (3)	O10B—H10B	0.8406
Mo6—O22	2.160 (3)	O29B—H29A	0.8695
Mo6—O23	1.697 (4)	O29B—H29B	0.8704
Mo6—O24	1.715 (3)	O43B—H43A	0.8413
Mo6—O25	2.038 (3)	O43B—H43B	0.8491
Mo7—O25	2.050 (3)	O1W—H1WA	0.8444
Mo7—O26	1.709 (3)	O1W—H1WB	0.8441
Mo7—O27	2.400 (3)	O2W—H2WA	0.8292
Mo7—O28	1.868 (3)	O2W—H2WB	0.8502
Mo7—O29	1.690 (4)	O3W—H3WA	0.8442
Mo7—O30	2.122 (3)	O3W—H3WB	0.8443
Mo8—O20	2.047 (3)	O4W—H4WA	0.8446
Mo8—O22	1.994 (3)	O4W—H4WB	0.8448
Mo8—O25	2.019 (3)	O5W—H5WA	0.8443
Mo8—O30	1.971 (3)	O5W—H5WB	0.8454
Mo8—O31	1.674 (3)	O6W—H6WA	0.8444
Mo8—O32	2.016 (3)	O6W—H6WB	0.8452
Mo8—O33	2.309 (3)	O7W—H7WA	0.8446
Mo9—O16	2.364 (3)	O7W—H7WB	0.8451
Mo9—O18	1.863 (3)	O8W—H8WA	0.8445
Mo9—O20	2.361 (3)	O8W—H8WB	0.8442
Mo9—O33	1.744 (3)	O9W—H9WA	0.8443
Mo9—O34	1.744 (3)	O9W—H9WB	0.8445
Mo9—O41	1.873 (3)	O10W—H10C	0.8440
Mo10—O13	2.021 (3)	O10W—H10D	0.8443
Mo10—O14	1.985 (3)	O11W—H11A	0.8440
Mo10—O16	2.056 (3)	O11W—H11B	0.8442
Mo10—O34	2.313 (3)	O12W—H12C	0.8452
Mo10—O35	1.666 (3)	O12W—H12D	0.8459
Mo10—O36	1.982 (3)	O13W—H13A	0.8442
Mo10—O37	2.034 (3)	O13W—H13B	0.8440
Mo11—O36	2.020 (3)	O14W—H14A	0.8440
Mo11—O37	2.086 (3)	O14W—H14B	0.8442
Mo11—O46^ii^	1.738 (3)	O15W—H15A	0.8448
Mo11—O61	1.840 (3)	O15W—H15B	0.8460
Mo11—O62	1.692 (3)	O16W—H16A	0.8440
Mo11—O63	2.445 (4)	O16W—H16B	0.8437
Mo12—O16	2.118 (3)	O17W—H17A	0.8447
Mo12—O37	1.980 (3)	O17W—H17B	0.8452
Mo12—O38	1.695 (3)	O18W—H18A	0.8442
Mo12—O39	1.737 (3)	O18W—H18B	0.8444
Mo12—O40	1.913 (3)	O19W—H19A	0.8449
Mo12—O41	2.276 (3)	O19W—H19B	0.8450
Mo13—O20	2.123 (3)	O20W—H20A	0.8440
Mo13—O32	1.988 (3)	O20W—H20B	0.8440
Mo13—O40	1.916 (3)	O21W—H21A	0.8299
Mo13—O41	2.252 (3)	O21W—H21B	0.8397
Mo13—O42	1.700 (3)	O22W—H22A	0.8440
Mo13—O56	1.739 (3)	O22W—H22B	0.8440
Mo14—O30	2.002 (3)	O23W—H23A	0.8432
Mo14—O32	2.077 (3)	O23W—H23B	0.8433
Mo14—O43	1.699 (4)	N1A—H1AA	0.880 (11)
Mo14—O44	2.418 (4)	N1A—H1AB	0.878 (11)
Mo14—O45	1.740 (3)	N1A—H1AC	0.878 (11)
Mo14—O55	1.815 (3)	N1A—H1AD	0.878 (11)
Mo15—O28	1.964 (3)	N2A—H2AA	0.882 (12)
Mo15—O45	2.193 (3)	N2A—H2AB	0.878 (12)
Mo15—O46	2.179 (3)	N2A—H2AC	0.876 (12)
Mo15—O47	1.700 (3)	N2A—H2AD	0.883 (11)
Mo15—O48	1.911 (3)	N3A—H3AA	0.871 (11)
Mo15—O49	1.709 (4)	N3A—H3AB	0.872 (12)
Mo16—O13^ii^	2.055 (3)	N3A—H3AC	0.875 (11)
Mo16—O36^ii^	2.109 (3)	N3A—H3AD	0.872 (11)
Mo16—O48	1.871 (3)	N4A—H4AA	0.880 (11)
Mo16—O50	1.713 (3)	N4A—H4AB	0.881 (12)
Mo16—O51	1.697 (3)	N4A—H4AC	0.883 (12)
Mo16—O52	2.465 (3)	N4A—H4AD	0.880 (11)
Mo17—O53	1.701 (4)	N5A—H5AA	0.873 (11)
Mo17—O54	1.701 (4)	N5A—H5AB	0.876 (12)
Mo17—O55	1.952 (3)	N5A—H5AC	0.877 (12)
Mo17—O56	2.291 (3)	N5A—H5AD	0.868 (12)
Mo17—O57	2.235 (4)	N6A—H6AA	0.8330
Mo17—O58	1.931 (3)	N6A—H6AB	0.7709
Mo18—O39	2.266 (3)	N6A—H6AC	0.9435
Mo18—O57	2.198 (3)	N6A—H6AD	0.7562
Mo18—O58	1.947 (3)	N7A—H7AA	0.877 (12)
Mo18—O59	1.700 (3)	N7A—H7AB	0.880 (12)
Mo18—O60	1.720 (3)	N7A—H7AC	0.880 (12)
Mo18—O61	1.961 (3)	N7A—H7AD	0.879 (12)
			
O1—Mo1—O2	105.05 (17)	O51—Mo16—O48	101.59 (15)
O1—Mo1—O3	99.13 (15)	O51—Mo16—O50	105.05 (17)
O1—Mo1—O4	92.26 (16)	O51—Mo16—O52	173.49 (15)
O1—Mo1—O5	99.13 (16)	O53—Mo17—O54	104.11 (19)
O1—Mo1—O19	171.02 (15)	O53—Mo17—O55	97.41 (16)
O2—Mo1—O3	98.95 (15)	O53—Mo17—O56	89.66 (15)
O2—Mo1—O4	161.09 (14)	O53—Mo17—O57	166.36 (15)
O2—Mo1—O5	95.08 (14)	O53—Mo17—O58	97.60 (15)
O2—Mo1—O19	83.87 (14)	O54—Mo17—O55	97.39 (16)
O3—Mo1—O4	85.49 (14)	O54—Mo17—O56	165.86 (16)
O3—Mo1—O5	153.18 (13)	O54—Mo17—O57	88.34 (16)
O3—Mo1—O19	78.08 (12)	O54—Mo17—O58	100.11 (16)
O4—Mo1—O19	79.06 (12)	O55—Mo17—O56	77.30 (13)
O5—Mo1—O4	74.32 (13)	O55—Mo17—O57	86.28 (14)
O5—Mo1—O19	80.85 (13)	O57—Mo17—O56	78.30 (12)
O4—Mo2—O9	79.35 (12)	O58—Mo17—O55	153.37 (14)
O5—Mo2—O4	73.41 (13)	O58—Mo17—O56	80.92 (12)
O5—Mo2—O9	81.59 (13)	O58—Mo17—O57	74.38 (13)
O6—Mo2—O4	94.42 (15)	O57—Mo18—O39	78.43 (11)
O6—Mo2—O5	99.05 (16)	O58—Mo18—O39	81.65 (13)
O6—Mo2—O7	103.81 (16)	O58—Mo18—O57	74.95 (13)
O6—Mo2—O8	98.95 (16)	O58—Mo18—O61	155.46 (13)
O6—Mo2—O9	173.32 (14)	O59—Mo18—O39	167.79 (13)
O7—Mo2—O4	160.37 (15)	O59—Mo18—O57	89.95 (14)
O7—Mo2—O5	96.23 (15)	O59—Mo18—O58	99.06 (16)
O7—Mo2—O8	100.07 (15)	O59—Mo18—O60	104.78 (16)
O7—Mo2—O9	82.69 (13)	O59—Mo18—O61	97.97 (15)
O8—Mo2—O4	83.98 (14)	O60—Mo18—O39	87.29 (14)
O8—Mo2—O5	152.00 (14)	O60—Mo18—O57	162.91 (15)
O8—Mo2—O9	78.12 (13)	O60—Mo18—O58	93.89 (15)
O8—Mo3—O12	79.01 (19)	O60—Mo18—O61	98.70 (15)
O8—Mo3—O13	148.45 (14)	O61—Mo18—O39	78.02 (12)
O8—Mo3—O14	85.61 (13)	O61—Mo18—O57	87.55 (13)
O10—Mo3—O8	100.72 (17)	O30—Mo1B—O32	66.4 (4)
O10—Mo3—O11	105.0 (2)	O30—Mo1B—O43B	78.6 (4)
O10—Mo3—O12	166.51 (18)	O32—Mo1B—O43B	78.7 (4)
O10—Mo3—O13	96.29 (15)	O45—Mo1B—O30	90.9 (5)
O10—Mo3—O14	93.82 (15)	O45—Mo1B—O32	152.9 (7)
O11—Mo3—O8	103.51 (17)	O45—Mo1B—O55	109.3 (6)
O11—Mo3—O12	88.0 (2)	O45—Mo1B—O43B	82.7 (4)
O11—Mo3—O13	97.39 (16)	O45—Mo1B—O44B	107.5 (6)
O11—Mo3—O14	156.87 (17)	O55—Mo1B—O30	148.3 (7)
O13—Mo3—O12	78.37 (18)	O55—Mo1B—O32	86.7 (5)
O13—Mo3—O14	66.80 (12)	O55—Mo1B—O43B	80.1 (4)
O14—Mo3—O12	72.70 (16)	O44B—Mo1B—O30	94.7 (5)
O9—Mo4—O14	91.56 (14)	O44B—Mo1B—O32	89.7 (4)
O9—Mo4—O16	156.54 (13)	O44B—Mo1B—O55	101.7 (6)
O9—Mo4—O17	101.64 (15)	O44B—Mo1B—O43B	168.1 (6)
O9—Mo4—O18	90.38 (13)	O8—Mo3B—O13	156.4 (3)
O14—Mo4—O16	69.90 (13)	O8—Mo3B—O14	90.9 (2)
O14—Mo4—O18	79.26 (12)	O8—Mo3B—O10B	84.4 (3)
O15—Mo4—O9	105.12 (15)	O11—Mo3B—O8	102.1 (3)
O15—Mo4—O14	102.08 (15)	O11—Mo3B—O13	94.0 (3)
O15—Mo4—O16	93.09 (14)	O11—Mo3B—O14	158.9 (4)
O15—Mo4—O17	100.75 (15)	O11—Mo3B—O10B	82.2 (4)
O15—Mo4—O18	164.35 (14)	O13—Mo3B—O14	69.07 (18)
O16—Mo4—O18	72.56 (11)	O13—Mo3B—O10B	80.9 (3)
O17—Mo4—O14	149.56 (13)	O14—Mo3B—O10B	82.6 (2)
O17—Mo4—O16	89.06 (13)	O12B—Mo3B—O8	104.0 (13)
O17—Mo4—O18	73.40 (12)	O12B—Mo3B—O11	87.7 (11)
O17—Mo5—O18	72.69 (12)	O12B—Mo3B—O13	93.6 (13)
O17—Mo5—O20	88.68 (12)	O12B—Mo3B—O14	105.4 (12)
O17—Mo5—O22	149.39 (13)	O12B—Mo3B—O10B	168.1 (12)
O19—Mo5—O17	100.87 (14)	O25—Mo7B—O30	65.9 (2)
O19—Mo5—O18	89.91 (14)	O25—Mo7B—O29B	78 (2)
O19—Mo5—O20	156.29 (14)	O26—Mo7B—O25	91.5 (4)
O19—Mo5—O22	92.01 (13)	O26—Mo7B—O28	106.4 (4)
O20—Mo5—O18	72.13 (12)	O26—Mo7B—O30	147.5 (6)
O21—Mo5—O17	100.15 (15)	O26—Mo7B—O29B	70.8 (17)
O21—Mo5—O18	163.96 (14)	O28—Mo7B—O25	151.1 (5)
O21—Mo5—O19	105.70 (17)	O28—Mo7B—O30	87.9 (3)
O21—Mo5—O20	93.71 (15)	O28—Mo7B—O29B	86 (2)
O21—Mo5—O22	102.91 (15)	O30—Mo7B—O29B	81.4 (18)
O22—Mo5—O18	79.75 (12)	O27B—Mo7B—O25	90.0 (4)
O22—Mo5—O20	70.08 (12)	O27B—Mo7B—O26	106.5 (5)
O3—Mo6—O2^i^	83.51 (14)	O27B—Mo7B—O28	105.8 (5)
O3—Mo6—O22	85.22 (13)	O27B—Mo7B—O30	96.9 (4)
O3—Mo6—O25	150.10 (14)	O27B—Mo7B—O29B	168 (2)
O22—Mo6—O2^i^	73.16 (12)	Mo1—O2—Mo6^i^	159.76 (19)
O23—Mo6—O2^i^	168.76 (15)	Mo6—O3—Mo1	157.7 (2)
O23—Mo6—O3	101.07 (17)	Mo1—O4—Mo2	95.77 (13)
O23—Mo6—O22	96.84 (15)	Mo1—O4—H4	112 (5)
O23—Mo6—O24	103.41 (18)	Mo2—O4—H4	123 (4)
O23—Mo6—O25	95.61 (15)	Mo1—O5—Mo2	113.32 (16)
O24—Mo6—O2^i^	85.12 (15)	Mo3—O8—Mo2	162.0 (2)
O24—Mo6—O3	104.60 (15)	Mo3B—O8—Mo2	163.3 (3)
O24—Mo6—O22	155.13 (15)	Mo4—O9—Mo2	153.34 (17)
O24—Mo6—O25	95.30 (14)	Mo3—O12—H12A	115.0
O25—Mo6—O2^i^	76.09 (13)	Mo3—O12—H12B	114.2
O25—Mo6—O22	68.18 (12)	H12A—O12—H12B	98.6
O25—Mo7—O27	73.88 (13)	Mo3—O13—Mo16^ii^	131.67 (16)
O25—Mo7—O30	67.58 (12)	Mo10—O13—Mo3	112.48 (15)
O26—Mo7—O25	93.55 (14)	Mo10—O13—Mo16^ii^	110.25 (14)
O26—Mo7—O27	81.98 (15)	Mo10—O13—Mo3B	109.6 (2)
O26—Mo7—O28	103.68 (15)	Mo4—O14—Mo3	140.82 (16)
O26—Mo7—O30	156.83 (15)	Mo4—O14—Mo10	111.45 (15)
O28—Mo7—O25	146.21 (14)	Mo4—O14—Mo3B	138.4 (2)
O28—Mo7—O27	79.96 (13)	Mo10—O14—Mo3	107.22 (14)
O28—Mo7—O30	87.29 (13)	Mo10—O14—Mo3B	109.06 (18)
O29—Mo7—O25	99.9 (3)	Mo4—O16—Mo9	99.45 (11)
O29—Mo7—O26	103.82 (18)	Mo10—O16—Mo4	102.58 (14)
O29—Mo7—O27	171.9 (2)	Mo10—O16—Mo9	96.67 (12)
O29—Mo7—O28	103.8 (2)	Mo10—O16—Mo12	104.88 (13)
O29—Mo7—O30	92.99 (17)	Mo12—O16—Mo4	143.48 (16)
O30—Mo7—O27	79.92 (12)	Mo12—O16—Mo9	100.78 (12)
O20—Mo8—O33	74.97 (11)	Mo4—O17—Mo5	114.59 (15)
O22—Mo8—O20	71.80 (12)	Mo4—O18—Mo5	90.92 (11)
O22—Mo8—O25	71.86 (12)	Mo9—O18—Mo4	112.90 (14)
O22—Mo8—O32	141.81 (12)	Mo9—O18—Mo5	112.61 (15)
O22—Mo8—O33	83.03 (12)	Mo5—O19—Mo1	152.15 (17)
O25—Mo8—O20	138.51 (13)	Mo5—O20—Mo9	100.19 (13)
O25—Mo8—O33	81.42 (12)	Mo8—O20—Mo5	102.86 (12)
O30—Mo8—O20	138.56 (12)	Mo8—O20—Mo9	97.09 (12)
O30—Mo8—O22	142.50 (13)	Mo8—O20—Mo13	105.05 (14)
O30—Mo8—O25	71.12 (12)	Mo13—O20—Mo5	143.15 (15)
O30—Mo8—O32	70.89 (12)	Mo13—O20—Mo9	99.73 (12)
O30—Mo8—O33	85.85 (12)	Mo5—O22—Mo6	140.75 (16)
O31—Mo8—O20	99.80 (14)	Mo5—O22—Mo8	110.67 (14)
O31—Mo8—O22	95.24 (15)	Mo8—O22—Mo6	107.79 (13)
O31—Mo8—O25	102.78 (14)	Mo6—O25—Mo7	135.73 (16)
O31—Mo8—O30	98.43 (15)	Mo6—O25—Mo7B	138.5 (2)
O31—Mo8—O32	96.15 (15)	Mo8—O25—Mo6	111.70 (14)
O31—Mo8—O33	174.77 (13)	Mo8—O25—Mo7	111.14 (14)
O32—Mo8—O20	70.38 (12)	Mo8—O25—Mo7B	108.7 (2)
O32—Mo8—O25	139.50 (12)	Mo7—O27—H27A	115.9
O32—Mo8—O33	82.35 (12)	Mo7—O27—H27B	114.4
O18—Mo9—O16	75.05 (11)	H27A—O27—H27B	106.4
O18—Mo9—O20	75.03 (13)	Mo7—O28—Mo15	152.03 (17)
O18—Mo9—O41	135.10 (13)	Mo7B—O28—Mo15	157.1 (3)
O20—Mo9—O16	93.79 (11)	Mo8—O30—Mo7	110.16 (14)
O33—Mo9—O16	172.95 (13)	Mo8—O30—Mo14	111.26 (14)
O33—Mo9—O18	103.57 (14)	Mo8—O30—Mo1B	113.6 (3)
O33—Mo9—O20	79.21 (12)	Mo8—O30—Mo7B	107.2 (2)
O33—Mo9—O34	107.35 (14)	Mo14—O30—Mo7	131.24 (15)
O33—Mo9—O41	102.70 (14)	Mo1B—O30—Mo7B	136.8 (4)
O34—Mo9—O16	79.66 (12)	Mo8—O32—Mo14	106.49 (13)
O34—Mo9—O18	102.97 (14)	Mo8—O32—Mo1B	108.1 (3)
O34—Mo9—O20	173.44 (12)	Mo13—O32—Mo8	111.54 (14)
O34—Mo9—O41	103.06 (14)	Mo13—O32—Mo14	140.51 (16)
O41—Mo9—O16	74.45 (12)	Mo13—O32—Mo1B	139.6 (3)
O41—Mo9—O20	74.96 (12)	Mo9—O33—Mo8	108.71 (14)
O13—Mo10—O16	140.87 (12)	Mo9—O34—Mo10	108.43 (13)
O13—Mo10—O34	86.91 (12)	Mo10—O36—Mo11	111.91 (15)
O13—Mo10—O37	142.45 (12)	Mo10—O36—Mo16^ii^	109.61 (14)
O14—Mo10—O13	71.73 (13)	Mo11—O36—Mo16^ii^	138.01 (15)
O14—Mo10—O16	71.61 (13)	Mo10—O37—Mo11	107.18 (14)
O14—Mo10—O34	82.50 (12)	Mo12—O37—Mo10	111.04 (15)
O14—Mo10—O37	141.46 (13)	Mo12—O37—Mo11	140.75 (16)
O16—Mo10—O34	75.23 (11)	Mo12—O39—Mo18	152.78 (17)
O35—Mo10—O13	98.53 (15)	Mo12—O40—Mo13	114.76 (15)
O35—Mo10—O14	97.26 (14)	Mo9—O41—Mo12	112.51 (14)
O35—Mo10—O16	99.18 (15)	Mo9—O41—Mo13	112.27 (14)
O35—Mo10—O34	174.21 (15)	Mo13—O41—Mo12	90.83 (11)
O35—Mo10—O36	102.66 (14)	Mo14—O44—H44A	110.5
O35—Mo10—O37	93.87 (14)	Mo14—O44—H44B	110.6
O36—Mo10—O13	71.58 (13)	H44A—O44—H44B	103.5
O36—Mo10—O14	140.29 (13)	Mo14—O45—Mo15	152.0 (2)
O36—Mo10—O16	136.50 (13)	Mo1B—O45—Mo15	138.9 (4)
O36—Mo10—O34	80.89 (11)	Mo11^ii^—O46—Mo15	154.54 (19)
O36—Mo10—O37	71.16 (13)	Mo16—O48—Mo15	161.31 (19)
O37—Mo10—O16	70.23 (13)	Mo16—O52—H52A	99.4
O37—Mo10—O34	82.89 (11)	Mo16—O52—H52B	101.1
O36—Mo11—O37	69.35 (12)	H52A—O52—H52B	109.4
O36—Mo11—O63	76.29 (13)	Mo14—O55—Mo17	156.2 (2)
O37—Mo11—O63	75.58 (12)	Mo1B—O55—Mo17	166.8 (4)
O46^ii^—Mo11—O36	91.21 (14)	Mo13—O56—Mo17	153.52 (17)
O46^ii^—Mo11—O37	149.88 (14)	Mo17—O57—H57	113 (4)
O46^ii^—Mo11—O61	103.59 (15)	Mo18—O57—Mo17	93.58 (12)
O46^ii^—Mo11—O63	77.59 (15)	Mo18—O57—H57	120 (4)
O61—Mo11—O36	152.82 (13)	Mo17—O58—Mo18	112.85 (16)
O61—Mo11—O37	87.36 (13)	Mo11—O61—Mo18	150.74 (19)
O61—Mo11—O63	84.68 (14)	Mo11—O63—H63A	127 (6)
O62—Mo11—O36	96.30 (14)	Mo11—O63—H63B	114 (5)
O62—Mo11—O37	99.94 (14)	H63A—O63—H63B	95 (7)
O62—Mo11—O46^ii^	105.06 (17)	Mo3B—O10B—H10A	119.2
O62—Mo11—O61	101.53 (16)	Mo3B—O10B—H10B	110.0
O62—Mo11—O63	172.27 (15)	H10A—O10B—H10B	116.9
O16—Mo12—O41	72.23 (11)	Mo7B—O29B—H29A	106.6
O37—Mo12—O16	69.96 (13)	Mo7B—O29B—H29B	113.5
O37—Mo12—O41	80.59 (12)	H29A—O29B—H29B	104.5
O38—Mo12—O16	94.02 (14)	Mo1B—O43B—H43A	119.7
O38—Mo12—O37	100.37 (15)	Mo1B—O43B—H43B	109.1
O38—Mo12—O39	104.46 (15)	H43A—O43B—H43B	104.6
O38—Mo12—O40	101.48 (15)	H1WA—O1W—H1WB	105.0
O38—Mo12—O41	165.12 (14)	H2WA—O2W—H2WB	109.3
O39—Mo12—O16	155.93 (13)	H3WA—O3W—H3WB	104.9
O39—Mo12—O37	91.33 (14)	H4WA—O4W—H4WB	104.8
O39—Mo12—O40	100.56 (15)	H5WA—O5W—H5WB	104.8
O39—Mo12—O41	90.33 (13)	H6WA—O6W—H6WB	104.9
O40—Mo12—O16	90.51 (13)	H7WA—O7W—H7WB	104.9
O40—Mo12—O37	151.57 (12)	H8WA—O8W—H8WB	104.9
O40—Mo12—O41	73.66 (12)	H9WA—O9W—H9WB	104.9
O20—Mo13—O41	73.02 (12)	H10C—O10W—H10D	91.8
O32—Mo13—O20	69.36 (12)	H11A—O11W—H11B	122.3
O32—Mo13—O41	80.95 (12)	H12C—O12W—H12D	104.8
O40—Mo13—O20	91.65 (12)	H13A—O13W—H13B	105.0
O40—Mo13—O32	152.36 (14)	H14A—O14W—H14B	104.9
O40—Mo13—O41	74.17 (12)	H15A—O15W—H15B	104.6
O42—Mo13—O20	93.47 (14)	H16A—O16W—H16B	105.1
O42—Mo13—O32	101.69 (15)	H17A—O17W—H17B	104.8
O42—Mo13—O40	99.27 (14)	H18A—O18W—H18B	104.9
O42—Mo13—O41	164.51 (14)	H19A—O19W—H19B	104.8
O42—Mo13—O56	105.02 (16)	H20A—O20W—H20B	105.0
O56—Mo13—O20	155.19 (13)	H21A—O21W—H21B	110.5
O56—Mo13—O32	90.45 (13)	H22A—O22W—H22B	105.0
O56—Mo13—O40	101.33 (14)	H23A—O23W—H23B	100.5
O56—Mo13—O41	90.14 (14)	H1AA—N1A—H1AB	109.1 (16)
O30—Mo14—O32	69.05 (12)	H1AA—N1A—H1AC	109.2 (16)
O30—Mo14—O44	78.58 (14)	H1AA—N1A—H1AD	105.3 (19)
O32—Mo14—O44	75.73 (13)	H1AB—N1A—H1AC	110 (4)
O43—Mo14—O30	99.27 (15)	H1AB—N1A—H1AD	111.5 (18)
O43—Mo14—O32	99.01 (15)	H1AC—N1A—H1AD	111.7 (18)
O43—Mo14—O44	174.72 (15)	H2AA—N2A—H2AB	109 (6)
O43—Mo14—O45	104.28 (18)	H2AA—N2A—H2AC	116 (6)
O43—Mo14—O55	101.18 (17)	H2AA—N2A—H2AD	109.2 (16)
O45—Mo14—O30	90.31 (14)	H2AB—N2A—H2AC	111.7 (19)
O45—Mo14—O32	151.12 (16)	H2AB—N2A—H2AD	105 (2)
O45—Mo14—O44	80.63 (16)	H2AC—N2A—H2AD	104.6 (19)
O45—Mo14—O55	103.52 (16)	H3AA—N3A—H3AB	110.6 (17)
O55—Mo14—O30	151.52 (15)	H3AA—N3A—H3AC	107 (2)
O55—Mo14—O32	88.26 (14)	H3AA—N3A—H3AD	110.8 (17)
O55—Mo14—O44	79.29 (16)	H3AB—N3A—H3AC	108 (5)
O28—Mo15—O45	83.31 (13)	H3AB—N3A—H3AD	108 (5)
O28—Mo15—O46	78.68 (13)	H3AC—N3A—H3AD	112.3 (19)
O46—Mo15—O45	75.71 (14)	H4AA—N4A—H4AB	105 (2)
O47—Mo15—O28	95.23 (15)	H4AA—N4A—H4AC	108.4 (17)
O47—Mo15—O45	168.89 (19)	H4AA—N4A—H4AD	109.2 (17)
O47—Mo15—O46	93.20 (18)	H4AB—N4A—H4AC	107 (6)
O47—Mo15—O48	98.20 (16)	H4AB—N4A—H4AD	110.6 (18)
O47—Mo15—O49	102.8 (2)	H4AC—N4A—H4AD	116 (6)
O48—Mo15—O28	156.76 (14)	H5AA—N5A—H5AB	107 (6)
O48—Mo15—O45	79.86 (13)	H5AA—N5A—H5AC	109.8 (18)
O48—Mo15—O46	81.70 (13)	H5AA—N5A—H5AD	111.0 (18)
O49—Mo15—O28	96.78 (17)	H5AB—N5A—H5AC	109 (6)
O49—Mo15—O45	88.28 (19)	H5AB—N5A—H5AD	113 (2)
O49—Mo15—O46	163.72 (19)	H5AC—N5A—H5AD	107 (6)
O49—Mo15—O48	98.61 (16)	H6AA—N6A—H6AB	96.2
O13^ii^—Mo16—O36^ii^	68.38 (12)	H6AA—N6A—H6AC	112.6
O13^ii^—Mo16—O52	80.67 (12)	H6AA—N6A—H6AD	122.3
O36^ii^—Mo16—O52	72.51 (12)	H6AB—N6A—H6AC	96.8
O48—Mo16—O13^ii^	152.32 (13)	H6AB—N6A—H6AD	122.7
O48—Mo16—O36^ii^	87.98 (13)	H6AC—N6A—H6AD	103.7
O48—Mo16—O52	78.50 (13)	H7AA—N7A—H7AB	106 (2)
O50—Mo16—O13^ii^	92.74 (15)	H7AA—N7A—H7AC	109.3 (17)
O50—Mo16—O36^ii^	149.54 (14)	H7AA—N7A—H7AD	109.6 (18)
O50—Mo16—O48	101.83 (15)	H7AB—N7A—H7AC	110.9 (19)
O50—Mo16—O52	81.21 (15)	H7AB—N7A—H7AD	111 (8)
O51—Mo16—O13^ii^	97.07 (15)	H7AC—N7A—H7AD	110 (8)
O51—Mo16—O36^ii^	100.99 (15)		
			
O1—Mo1—O2—Mo6^i^	−118.9 (5)	O30—Mo1B—O45—Mo15	62.3 (7)
O2^i^—Mo6—O3—Mo1	−139.2 (5)	O30—Mo1B—O55—Mo17	−55 (2)
O3—Mo1—O2—Mo6^i^	−16.9 (5)	O30—Mo7B—O28—Mo15	−26.4 (13)
O4—Mo1—O2—Mo6^i^	85.5 (7)	O32—Mo13—O56—Mo17	−22.6 (4)
O5—Mo1—O2—Mo6^i^	140.2 (5)	O32—Mo14—O45—Mo15	−29.7 (7)
O10—Mo3—O8—Mo2	−40.3 (7)	O32—Mo14—O55—Mo17	58.2 (6)
O11—Mo3—O8—Mo2	−148.8 (6)	O32—Mo1B—O45—Mo15	94.0 (14)
O11—Mo3B—O8—Mo2	174.2 (9)	O32—Mo1B—O55—Mo17	−24 (2)
O12—Mo3—O8—Mo2	126.0 (7)	O33—Mo9—O18—Mo4	−174.24 (15)
O13—Mo3—O8—Mo2	81.1 (7)	O33—Mo9—O18—Mo5	−73.01 (16)
O13^ii^—Mo16—O48—Mo15	48.0 (7)	O33—Mo9—O34—Mo10	−179.67 (13)
O13—Mo3B—O8—Mo2	−54 (2)	O33—Mo9—O41—Mo12	174.77 (15)
O14—Mo3—O8—Mo2	52.8 (7)	O33—Mo9—O41—Mo13	74.01 (16)
O14—Mo4—O9—Mo2	−17.1 (4)	O34—Mo9—O18—Mo4	74.02 (17)
O14—Mo3B—O8—Mo2	−22.6 (13)	O34—Mo9—O18—Mo5	175.25 (14)
O15—Mo4—O9—Mo2	−120.0 (4)	O34—Mo9—O33—Mo8	178.95 (13)
O16—Mo4—O9—Mo2	19.7 (6)	O34—Mo9—O41—Mo12	−73.76 (16)
O16—Mo9—O18—Mo4	−1.37 (13)	O34—Mo9—O41—Mo13	−174.52 (14)
O16—Mo9—O18—Mo5	99.85 (15)	O36—Mo11—O61—Mo18	−95.9 (5)
O16—Mo9—O34—Mo10	1.12 (12)	O36^ii^—Mo16—O48—Mo15	17.4 (6)
O16—Mo9—O41—Mo12	1.42 (13)	O37—Mo11—O61—Mo18	−65.5 (4)
O16—Mo9—O41—Mo13	−99.34 (15)	O37—Mo12—O39—Mo18	19.2 (4)
O16—Mo12—O39—Mo18	−18.7 (6)	O38—Mo12—O39—Mo18	120.3 (4)
O17—Mo4—O9—Mo2	135.3 (4)	O40—Mo12—O39—Mo18	−134.8 (4)
O17—Mo5—O19—Mo1	−139.0 (4)	O40—Mo13—O56—Mo17	132.3 (4)
O18—Mo4—O9—Mo2	62.2 (4)	O41—Mo9—O18—Mo4	−50.0 (2)
O18—Mo5—O19—Mo1	−66.7 (4)	O41—Mo9—O18—Mo5	51.2 (2)
O18—Mo9—O33—Mo8	70.45 (16)	O41—Mo9—O33—Mo8	−72.81 (16)
O18—Mo9—O34—Mo10	−70.75 (15)	O41—Mo9—O34—Mo10	72.33 (15)
O18—Mo9—O41—Mo12	50.2 (2)	O41—Mo12—O39—Mo18	−61.4 (4)
O18—Mo9—O41—Mo13	−50.5 (2)	O41—Mo13—O56—Mo17	58.4 (4)
O19—Mo1—O2—Mo6^i^	60.0 (5)	O42—Mo13—O56—Mo17	−124.8 (4)
O20—Mo5—O19—Mo1	−26.8 (6)	O43—Mo14—O45—Mo15	113.1 (5)
O20—Mo9—O18—Mo4	−99.55 (15)	O43—Mo14—O55—Mo17	−40.6 (6)
O20—Mo9—O18—Mo5	1.68 (11)	O44—Mo14—O45—Mo15	−65.0 (5)
O20—Mo9—O33—Mo8	−1.10 (12)	O44—Mo14—O55—Mo17	134.0 (6)
O20—Mo9—O41—Mo12	99.80 (15)	O45—Mo14—O55—Mo17	−148.4 (5)
O20—Mo9—O41—Mo13	−0.96 (12)	O45—Mo1B—O55—Mo17	178.5 (14)
O20—Mo13—O56—Mo17	12.0 (7)	O46^ii^—Mo11—O61—Mo18	142.9 (4)
O21—Mo5—O19—Mo1	117.0 (4)	O50—Mo16—O48—Mo15	168.3 (6)
O22—Mo5—O19—Mo1	13.0 (4)	O51—Mo16—O48—Mo15	−83.4 (6)
O22—Mo6—O3—Mo1	−65.6 (5)	O52—Mo16—O48—Mo15	89.9 (6)
O23—Mo6—O3—Mo1	30.5 (6)	O55—Mo14—O45—Mo15	−141.5 (5)
O24—Mo6—O3—Mo1	137.6 (5)	O55—Mo1B—O45—Mo15	−142.6 (4)
O25—Mo6—O3—Mo1	−92.2 (6)	O62—Mo11—O61—Mo18	34.1 (4)
O25—Mo7—O28—Mo15	81.6 (5)	O63—Mo11—O61—Mo18	−141.3 (4)
O25—Mo7B—O28—Mo15	−51 (2)	O10B—Mo3B—O8—Mo2	−105.0 (11)
O26—Mo7—O28—Mo15	−159.8 (4)	O12B—Mo3B—O8—Mo2	83.6 (15)
O26—Mo7B—O28—Mo15	−176.7 (6)	O27B—Mo7B—O28—Mo15	70.2 (11)
O27—Mo7—O28—Mo15	121.1 (4)	O29B—Mo7B—O28—Mo15	−108 (2)
O29—Mo7—O28—Mo15	−51.6 (4)	O43B—Mo1B—O45—Mo15	140.6 (4)
O30—Mo7—O28—Mo15	40.8 (4)	O43B—Mo1B—O55—Mo17	−102.9 (17)
O30—Mo14—O45—Mo15	13.4 (5)	O44B—Mo1B—O45—Mo15	−33.0 (10)
O30—Mo14—O55—Mo17	94.5 (6)	O44B—Mo1B—O55—Mo17	65 (2)

Symmetry codes: (i) −*x*+1, −*y*+2, −*z*+1; (ii) −*x*+1, −*y*+1, −*z*+1.

### Dodecaammoniumhexacosahydrate Hydrogen-bond geometry (Å, º)

**Table:**

*D*—H···*A*	*D*—H	H···*A*	*D*···*A*	*D*—H···*A*
O4—H4···O18	0.83 (2)	2.35 (3)	3.123 (5)	156 (6)
O12^a^—H12A^a^···O7W^a^^iii^	0.86	1.86	2.651 (8)	151
O12^a^—H12B^a^···O47^iii^	0.85	2.05	2.885 (7)	166
O12^a^—H12B^a^···O49^iii^	0.85	2.38	2.889 (7)	119
O27^a^—H27A^a^···O7W^a^	0.86	1.92	2.729 (6)	157
O27^a^—H27B^a^···O31	0.86	2.39	3.162 (5)	150
O44^a^—H44A^a^···O49	0.89	2.48	3.051 (6)	122
O44^a^—H44A^a^···O17W^a^	0.89	2.43	3.077 (11)	129
O44^a^—H44B^a^···O19W^a^	0.89	2.12	2.875 (7)	141
O52—H52*A*···O63^ii^	0.84	2.23	3.064 (5)	174
O52—H52*B*···O7^iv^	0.83	1.87	2.701 (5)	176
O57—H57···O41	0.82 (2)	2.18 (3)	2.981 (5)	163 (6)
O63—H63*A*···O12*W*^v^	0.92 (11)	1.96 (11)	2.818 (6)	155 (9)
O63—H63*B*···O6W^a^^v^	0.88 (8)	1.99 (7)	2.775 (7)	148 (7)
O63—H63*B*···O9W^b^^v^	0.88 (8)	1.89 (10)	2.71 (6)	155 (7)
O10B^b^—H10A^b^···O34	0.86	2.18	2.947 (5)	149
O10B^b^—H10B^b^···O5*W*	0.84	2.55	3.385 (7)	172
O29B^b^—H29A^b^···O2W^b^	0.87	2.62	3.34 (8)	141
O43B^b^—H43A^b^···O57	0.84	2.60	3.266 (5)	137
O43B^b^—H43A^b^···N5*A*^ii^	0.84	2.51	3.268 (6)	151
O43B^b^—H43B^b^···O33	0.85	2.08	2.901 (5)	161
O1W^a^—H1WA^a^···O10^a^	0.84	1.96	2.772 (7)	160
O1W^a^—H1WA^a^···O34	0.84	2.65	3.180 (6)	122
O1W^a^—H1WB^a^···O23	0.84	2.12	2.900 (7)	153
O2W^b^—H2WB^b^···O23	0.85	1.92	2.76 (3)	170
O3W^b^—H3WA^b^···O23	0.84	2.63	3.17 (2)	123
O3W^b^—H3WA^b^···O2W^b^	0.84	1.70	2.51 (4)	160
O3W^b^—H3WB^b^···O5*W*	0.84	2.07	2.80 (2)	145
O4W^a^—H4WA^a^···O23	0.84	2.54	3.262 (7)	144
O4W^a^—H4WB^a^···O6W^a^^i^	0.84	2.38	2.917 (12)	122
O5*W*—H5*WA*···O4	0.84	2.18	2.827 (6)	133
O5*W*—H5*WB*···O10^a^	0.85	2.65	3.385 (7)	145
O6W^a^—H6WA^a^···O24^i^	0.84	2.30	2.866 (7)	125
O6W^a^—H6WB^a^···O21	0.85	2.43	3.164 (9)	146
O7W^a^—H7WA^a^···O52^vi^	0.84	2.06	2.819 (6)	149
O7W^a^—H7WB^a^···O17W^a^	0.85	1.87	2.672 (9)	159
O8W^b^—H8WA^b^···O35	0.84	2.39	3.02 (2)	132
O8W^b^—H8WB^b^···O49^iii^	0.84	2.08	2.834 (19)	149
O8W^b^—H8WB^b^···O18W^b^^iii^	0.84	2.48	2.95 (3)	117
O9W^b^—H9WA^b^···O24^i^	0.84	2.22	2.79 (6)	125
O9W^b^—H9WA^b^···N1*A*^i^	0.84	2.65	3.42 (8)	152
O9W^b^—H9WB^b^···O21	0.84	2.21	2.95 (7)	146
O10W^a^—H10C^a^···O61^vii^	0.84	2.26	3.036 (6)	153
O10W^a^—H10D^a^···O17	0.84	2.18	2.883 (6)	140
O11W^b^—H11A^b^···O17	0.84	2.38	2.98 (3)	128
O11W^b^—H11A^b^···O40	0.84	2.59	3.01 (3)	112
O11W^b^—H11B^b^···O61^vii^	0.84	2.41	3.22 (3)	162
O12*W*—H12*C*···O14W^b^	0.85	2.05	2.852 (14)	158
O12*W*—H12*C*···O15W^a^	0.85	1.99	2.765 (11)	151
O12*W*—H12*D*···O6^viii^	0.85	2.50	3.248 (6)	148
O14W^b^—H14B^b^···O21	0.84	2.23	3.043 (15)	160
O15W^a^—H15A^a^···O21	0.84	2.37	3.165 (11)	158
O15W^a^—H15B^a^···O31	0.85	2.35	3.076 (11)	144
O16W^a^—H16A^a^···O1^i^	0.84	2.43	3.017 (10)	128
O16W^a^—H16B^a^···O15W^a^	0.84	2.26	2.828 (14)	125
O17W^a^—H17A^a^···O4W^a^^viii^	0.84	2.14	2.857 (12)	143
O18W^b^—H18A^b^···O44B^b^	0.84	1.76	2.55 (3)	154
O18W^b^—H18B^b^···O27B^b^	0.84	2.15	2.84 (2)	139
O19W^a^—H19A^a^···O11^viii^	0.84	2.42	3.170 (7)	149
O19W^a^—H19A^a^···O42	0.84	2.43	2.919 (7)	118
O19W^a^—H19B^a^···O15W^a^	0.85	2.51	3.253 (14)	147
O20W^b^—H20A^b^···O8W^b^^ix^	0.84	2.14	2.97 (4)	167
O20W^b^—H20B^b^···O54^ii^	0.84	2.10	2.81 (3)	142
O20Wb—H20B^b^···O21*W*^x^	0.84	2.33	2.87 (3)	122
O21*W*—H21*A*···O45	0.83	2.33	3.144 (6)	169
O21*W*—H21*B*···O53^xi^	0.84	2.04	2.862 (5)	167
O22W^b^—H22A^b^···O50^vi^	0.84	2.43	2.84 (2)	111
O22W^b^—H22A^b^···O52^vi^	0.84	2.28	2.88 (3)	128
O22W^b^—H22B^b^···O27B^b^	0.84	1.86	2.70 (2)	175
O23*W*—H23*A*···O50^xi^	0.84	1.94	2.775 (5)	169
O23*W*—H23*B*···O39	0.84	2.55	3.143 (5)	128
O23*W*—H23*B*···O56	0.84	2.33	3.053 (5)	144
O23*W*—H23*B*···O58	0.84	2.31	2.977 (5)	137
N1*A*—H1*AA*···O60^ix^	0.88 (1)	2.00 (2)	2.860 (6)	166 (4)
N1*A*—H1*AB*···O24	0.88 (1)	2.08 (2)	2.931 (6)	164 (5)
N1*A*—H1*AB*···O26	0.88 (1)	2.60 (5)	3.073 (6)	115 (4)
N1*A*—H1*AC*···O23*W*^ix^	0.88 (1)	1.99 (2)	2.837 (6)	163 (5)
N1*A*—H1*AD*···O5^i^	0.88 (1)	1.95 (1)	2.827 (6)	174 (5)
N2*A*—H2*AA*···O3W^b^	0.88 (1)	1.95 (3)	2.811 (19)	165 (7)
N2*A*—H2*AA*···O4W^a^	0.88 (1)	2.03 (3)	2.848 (8)	153 (6)
N2*A*—H2*AB*···O29^a^	0.88 (1)	2.29 (4)	2.975 (6)	135 (4)
N2*A*—H2*AB*···O59^ii^	0.88 (1)	2.19 (4)	2.770 (6)	123 (4)
N2*A*—H2*AB*···O29B^b^	0.88 (1)	2.03 (7)	2.67 (6)	130 (4)
N2*A*—H2*AB*···O2W^b^	0.88 (1)	2.62 (6)	3.06 (3)	112 (4)
N2*A*—H2*AC*···O26	0.88 (1)	2.32 (3)	3.063 (6)	143 (4)
N2*A*—H2*AC*···O38^ix^	0.88 (1)	2.16 (4)	2.744 (6)	123 (4)
N2*A*—H2*AD*···O15^ix^	0.88 (1)	2.24 (2)	3.089 (6)	163 (4)
N3*A*—H3*AA*···O58^vii^	0.87 (1)	1.94 (2)	2.797 (5)	166 (4)
N3*A*—H3*AB*···O49^iii^	0.87 (1)	2.40 (4)	3.018 (6)	128 (4)
N3*A*—H3*AB*···O21*W*^iii^	0.87 (1)	2.21 (3)	2.989 (6)	149 (4)
N3*A*—H3*AC*···O7	0.88 (1)	2.01 (2)	2.873 (5)	170 (5)
N3*A*—H3*AD*···O15	0.87 (1)	2.26 (2)	3.107 (6)	164 (5)
N4*A*—H4*AA*···O40	0.88 (1)	2.38 (4)	2.924 (5)	120 (4)
N4*A*—H4*AA*···O10W^a^	0.88 (1)	2.09 (3)	2.910 (8)	154 (5)
N4*A*—H4*AA*···O11W^b^	0.88 (1)	1.78 (5)	2.47 (3)	134 (5)
N4*A*—H4*AB*···O23*W*	0.88 (1)	1.99 (1)	2.867 (7)	173 (6)
N4*A*—H4*AC*···O28^xii^	0.88 (1)	2.04 (2)	2.907 (5)	165 (5)
N4*A*—H4*AC*···O47^xii^	0.88 (1)	2.48 (6)	2.986 (6)	117 (5)
N4*A*—H4*AD*···O42	0.88 (1)	2.45 (5)	3.005 (5)	122 (4)
N4*A*—H4*AD*···O12*W*	0.88 (1)	2.24 (3)	2.967 (7)	140 (5)
N5*A*—H5*AA*···O57^ii^	0.87 (1)	1.96 (1)	2.830 (5)	177 (5)
N5*A*—H5*AB*···O62	0.88 (1)	2.45 (3)	3.232 (6)	149 (5)
N5*A*—H5*AC*···O1W^a^	0.88 (1)	1.96 (2)	2.824 (8)	170 (7)
N5*A*—H5*AC*···O2W^b^	0.88 (1)	1.94 (4)	2.81 (3)	167 (6)
N5*A*—H5*AD*···O43^a^	0.87 (1)	2.09 (2)	2.932 (6)	162 (5)
N6A^b^—H6AA^b^···O53	0.83	1.89	2.71 (3)	168
N6A^b^—H6AB^b^···O44B^b^	0.77	2.28	2.96 (3)	147
N6A^b^—H6AC^b^···O20W^b^^viii^	0.94	2.00	2.92 (4)	163
N6A^b^—H6AD^b^···O11^viii^	0.76	2.09	2.73 (3)	143
N6A^b^—H6AD^b^···O10B^b^^viii^	0.76	2.64	3.16 (3)	127
N7A^a^—H7AA^a^···O21*W*^x^	0.88 (1)	2.11 (2)	2.979 (8)	173 (6)
N7A^a^—H7AB^a^···O51^ii^	0.88 (1)	2.26 (4)	2.994 (7)	141 (6)
N7A^a^—H7AB^a^···O53^x^	0.88 (1)	2.27 (5)	2.948 (7)	134 (6)
N7A^a^—H7AC^a^···O1W^a^	0.88 (1)	2.08 (2)	2.945 (10)	168 (6)
N7A^a^—H7AD^a^···O19W^a^^x^	0.88 (1)	1.99 (2)	2.865 (9)	174 (9)

Symmetry codes: (i) −*x*+1, −*y*+2, −*z*+1; (ii) −*x*+1, −*y*+1, −*z*+1; (iii) *x*−1/2, −*y*+3/2, *z*+1/2; (iv) *x*+1/2, −*y*+3/2, *z*−1/2; (v) −*x*+3/2, *y*−1/2, −*z*+3/2; (vi) −*x*+3/2, *y*+1/2, −*z*+1/2; (vii) −*x*+3/2, *y*+1/2, −*z*+3/2; (viii) *x*+1, *y*, *z*; (ix) *x*−1/2, −*y*+3/2, *z*−1/2; (x) *x*−1, *y*, *z*; (xi) −*x*+2, −*y*+1, −*z*+1; (xii) *x*+1/2, −*y*+3/2, *z*+1/2.

## References

[bb1] Armour, A. W., Drew, M. G. B. & Mitchell, P. C. H. (1975). *J. Chem. Soc. Dalton Trans.* pp. 1493–1496..

[bb2] Atencio, R., Briceño, A. & Galindo, X. (2005). *Chem. Commun.* pp. 637–639. 10.1039/b413825b 15672161

[bb3] Baizig, J., Haj Abdallah, A. & Haddad, A. (2018). *Turk. J. Chem.* **42**, 317–331.

[bb4] Bruker (2012). *TWINABS*. Bruker AXS Inc., Madison, Wisconsin, USA.

[bb5] Bruker (2016). *APEX6* and *SAINT*. Bruker AXS Inc., Madison, Wisconsin, USA.

[bb6] Dolomanov, O. V., Bourhis, L. J., Gildea, R. J., Howard, J. A. K. & Puschmann, H. (2009). *J. Appl. Cryst.* **42**, 339–341.

[bb7] Eda, K. & Iriki, Y. (2005). *Chem. Lett.* **34**, 612–613.

[bb8] Falaise, C., Moussawi, M. A., Floquet, S., Abramov, P. A., Sokolov, M. N., Haouas, M. & Cadot, E. (2018). *J. Am. Chem. Soc.* **140**, 11198–11201. 10.1021/jacs.8b07525 30156413

[bb9] Gao, Q., Xu, L., Gong, J.-Y. & Wang, W.-N. (2019). *J. Cluster Sci.* **30**, 1065–1069.

[bb10] Garin, J. L. & Costamagna, J. A. (1988). *Acta Cryst.* C**44**, 779–782.

[bb11] Gatehouse, B. M. & Leverett, P. (1971). *J. Chem. Soc. A* pp. 2107–2112.

[bb12] Groom, C. R., Bruno, I. J., Lightfoot, M. P. & Ward, S. C. (2016). *Acta Cryst.* B**72**, 171–179. 10.1107/S2052520616003954 PMC4822653 27048719

[bb13] Gumerova, N. I. & Rompel, A. (2020). *Chem. Soc. Rev.* **49**, 7568–7601. 10.1039/d0cs00392a 32990698

[bb14] Himeno, S., Niiya, H. & Ueda, T. (1997). *Bull. Chem. Soc. Jpn* **70**, 631–637.

[bb15] Long, D.-L., Streb, C., Kögerler, P. & Cronin, L. (2006). *J. Cluster Sci.* **17**, 257–266.

[bb16] Ma, J., Li, Y.-G., Zhang, Z.-M., Wu, Q. & Wang, E.-B. (2009). *Inorg. Chim. Acta* **362**, 2413–2417.

[bb17] Paulat-Böschen, I. (1979). *J. Chem. Soc. Chem. Commun.* pp. 780–782.

[bb18] Priya, L., Unnikrishnan, P. M., Bommakanti, S., Pallepogu, R. & Das, S. K. (2026). *Inorg. Chem.* **65**, 15340–15345. 10.1021/acs.inorgchem.6c00623 42383689

[bb19] Román, P., Gutiérrez-Zorrilla, J. M., Luque, A. & Martínez-Ripoll, M. (1988). *J. Crystallogr. Spectrosc. Res.* **18**, 117–131.

[bb20] Sheldrick, G. M. (2015*a*). *Acta Cryst.* A**71**, 3–8.

[bb21] Sheldrick, G. M. (2015*b*). *Acta Cryst.* C**71**, 3–8.

[bb22] Spek, A. L. (2015). *Acta Cryst.* C**71**, 9–18. 10.1107/S2053229614024929 25567569

[bb23] Sukmana, N. C., Sugiarto, Shinogi, J., Minato, T., Kojima, T., Fujibayashi, M., Nishihara, S., Inoue, K., Cao, Y., Zhu, T., Ubukata, H., Higashiura, A., Yamamoto, A., Tassel, C., Kageyama, H., Sakaguchi, T. & Sadakane, M. (2024). *Inorg. Chem.* **63**, 10207–10220. 10.1021/acs.inorgchem.4c00543 38767574

